# Elastic and Self-Healing Copolymer Coatings with Antimicrobial Function

**DOI:** 10.1021/acsami.4c00431

**Published:** 2024-04-29

**Authors:** Livy Laysandra, Randy Arthur Rusli, Yu-Wei Chen, Shi-Ju Chen, Yao-Wei Yeh, Tsung-Lin Tsai, Jui-Hsiung Huang, Kao-Shu Chuang, Andreas Njotoprajitno, Yu-Cheng Chiu

**Affiliations:** † Department of Chemical Engineering, National Taiwan University of Science and Technology, Taipei 10607, Taiwan; ‡ Taipei Municipal Zhongshan Girls High School, Taipei 10617, Taiwan; § Department of Biomedical Engineering, College of Engineering, National Cheng Kung University, Tainan 704, Taiwan; ∥ Department of Oncology, National Cheng Kung University Hospital, College of Medicine, National Cheng Kung University, Tainan 704, Taiwan; ⊥ Department of Green Material Technology, Green Technology Research Institute, CPC Corporation, Kaohsiung City 811, Taiwan; ¶ Advanced Research Center for Green Materials Science and Technology, National Taiwan University, Taipei 10617, Taiwan

**Keywords:** amphiphilic copolymer, synergistic effect, antimicrobial, antifouling, self-healing, transparent

## Abstract

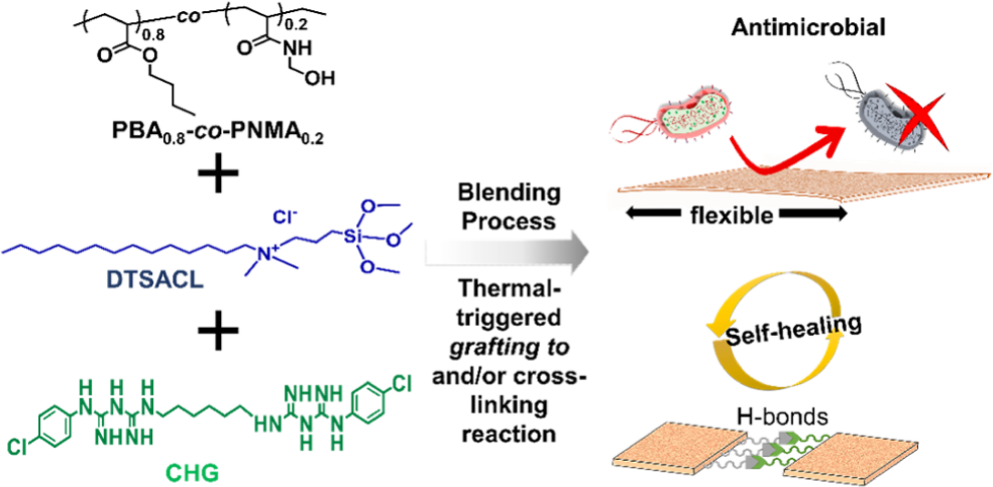

The revolutionary self-healing function for long-term and safe service processes has inspired researchers to implement them in various fields, including in the application of antimicrobial protective coatings. Despite the great advances that have been made in the field of fabricating self-healing and antimicrobial polymers, their poor transparency and the trade-off between the mechanical and self-healing properties limit the utility of the materials as transparent antimicrobial protective coatings for wearable optical and display devices. Considering the compatibility in the blending process, our group proposed a self-healing, self-cross-linkable poly{(*n*-butyl acrylate)-*co*-[*N*-(hydroxymethyl)acrylamide]} copolymer (AP)-based protective coating combined with two types of commercial cationic antimicrobial agents (i.e., dimethyl octadecyl (3-trimethoxysilylpropyl) ammonium chloride (DTSACL) and chlorhexidine gluconate (CHG)), leading to the fabrication of a multifunctional modified compound film of (AP/*b*%CHG)-*grafted*-*a*%DTSACL. The first highlight of this research is that the reactivity of the hydroxyl group in the *N*-(hydroxymethyl)acrylamide of the copolymer side chains under thermal conditions facilitates the “grafting to” process with the trimethoxysilane groups of DTSACL to form AP-*grafted*-DTSACL, yielding favorable thermal stability, improvement in hydrophobicity, and enhancement of mechanical strength. Second, we highlight that the addition of CHG can generate covalent and noncovalent interactions in a complex manner between the two biguanide groups of CHG with the AP and DTSACL via a thermal-triggered cross-linking reaction. The noncovalent interactions synergistically serve as diverse dynamic hydrogen bonds, leading to complete healing upon scratches and even showing over 80% self-healing efficiency on full-cut, while covalent bonding can effectively improve elasticity and mechanical strength. The soft nature of CHG also takes part in improving the self-healing of the copolymer. Moreover, it was discovered that the addition of CHG can enhance antimicrobial effectiveness, as demonstrated by the long-term superior antibacterial activity (100%) against Gram-negative (*Escherichia coli*) and Gram-positive (*Staphylococcus aureus*) bacteria and the antifouling function on a glass substrate and/or a silica wafer coated by the modified polymer.

## Introduction

The continuous accumulation of undesirable microorganisms and viruses deposited on various surfaces and further exacerbated by escalating antimicrobial resistance present precarious stages of forming a biofilm, posing a severe threat to global public health.^1−5^ Preventing biofilm formation through the development of novel antimicrobial agents that are highly effective as protective thin coatings has become a promising approach.^6^ A number of antimicrobial agents have been commercialized and applied as antibacterial and antifouling coatings in petroleum pipelines and aquatic flow systems,^7^ medical devices or implants,^8,9^ dental surgery equipment,^10,11^ marine vessels,^12,13^ textiles,^14,15^ and even on optical and display devices.^16^ More concrete examples of the practical antimicrobial coating applications are provided in Table S1. Two other well-known types of commercial antimicrobial agents are dimethyl octadecyl (3-trimethoxysilylpropyl) ammonium chloride (DTSACL) and chlorhexidine gluconate (CHG). The unique structure of DTSACL is described as having a quaternary ammonium group that is sequestered by a propylene spacer from a hydrophilic trimethoxysilane group and bound to an aliphatic octadecyl chain (C_26_H_58_NO_3_SiCl), which is responsible for its hydrophobicity.^17,18^ CHG, a nonoxidizing biocide, is a biguanide and cation-active compound that consists of two symmetric 4-chlorophenyl rings and two biguanide groups connected by a central hexamethylene chain.^19−22^ CHG has a broad spectrum of efficacy in the field of inhibiting microorganism adherence capability and preventing biofilm formation compared to other antimicrobials or biocides.^19,22,23^ Both DTSACL and CHG are also known as surface cationic contact killing surfaces, which have the same mechanism in eliminating various species of bacteria including Gram-negative bacteria and Gram-positive bacteria. The mechanism begins with the positively charged hydrophobic and lipophilic molecules in DTSACL or CHG interacting with negatively charged phospholipids and lipopolysaccharides on bacterial cell membranes. Then, they enter the cell via some type of active or passive transport mechanism based on electrostatic attraction and ionic interactions, thereby altering the cell’s osmotic equilibrium and leading to the consequent death of the bacteria.

Although antimicrobial agents have been applied as protective coatings, a current problem that arises in long-term practice is that the accumulation of dead bacteria adhering to surfaces could block the active antimicrobial sites, resulting in the loss of bactericidal efficacy and ultimately the failure to prevent biofilm formation. Amphiphilic copolymers induced by appropriate hydrophobic and hydrophilic balance have been developed by scientists as an alternative strategy for bacterial antiadhesion since such polymers can influence antimicrobial activity and selectivity.^24−27^ Particularly, amphiphilic surface coatings are often employed to improve fouling reduction under marine conditions. The hydrophilic domain proceeds as cell binders, while the hydrophobic domain serves as the bacterial cell wall penetration and inserts into the membrane. For instance, Du’s group had proposed novel poly(ethylene oxide)-*block*-poly(ε-caprolactone)-*block*-poly[(2-*tert*-butyl aminoethyl) methacrylate] amphiphilic ABC triblock copolymers that self-assemble into micelles in aqueous media, resulting in good antimicrobial efficacy (99.9%) against *Escherichia coli* and *Staphylococcus aureus* without loading any external antibiotics.^25^ Zhao’s group compared the antifouling performance based on differences in molecular architectures, where fluorinated amphiphilic copolymers (poly(hydroxyethyl methacrylate)-*r*-poly(2-perfluorooctyl methacrylate)s) exhibited better antifouling properties than the corresponding homopolymers.^26^

To complement long-term functionality, researchers seek to introduce an autonomous self-healing feature into antimicrobial polymer coatings to prevent surface degradation, crack, and abrasion, which can generate a rough morphology on the damaged material surface and eventually lead to fatal bacterial invasion.^28−30^ Great progress has been made in the synthesis of novel self-healable antimicrobial polymers; however, most synthetic procedures still entail the involvement of external stimuli to promote the healing process and cause inescapable colors to the resulting materials, thereby limiting the utility of the materials as transparent protective coatings.^16,28−34^ Although a direct combination of a self-healing polymer with an antimicrobial agent via a systematic blending is the most handy process, the number of studies is still scarce because the introduction of antimicrobial agents complicates both the uniformity due to the aggregation problem and the controllability of physical and mechanical properties as well as limits the room-temperature self-healing ability of the polymers.^35−37^ In this regard, selecting additional antimicrobial agents having compatibility with the host room-temperature self-healing polymer is key to alleviating the aforementioned issues.

**Scheme 1:**
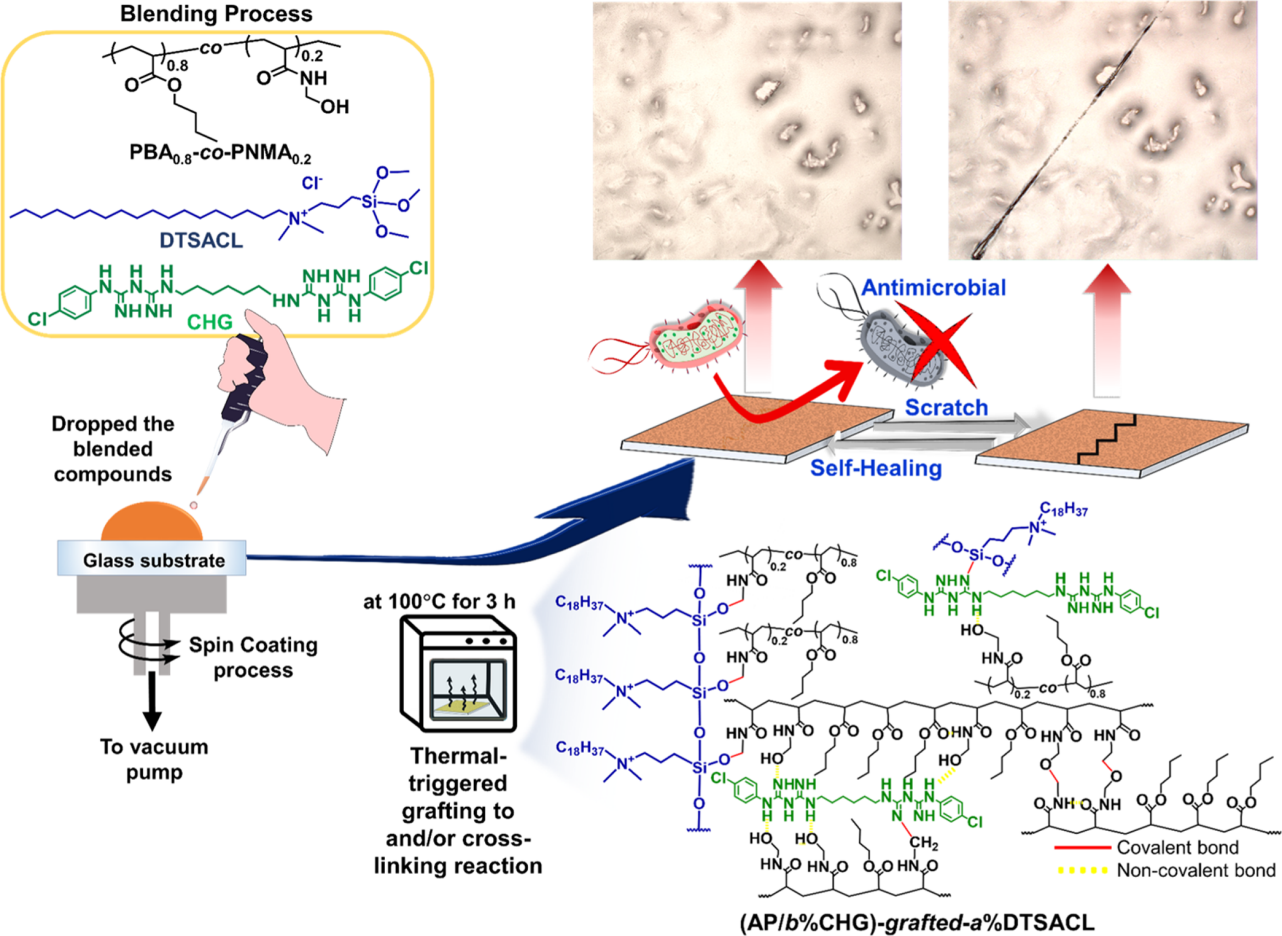
Schematic Illustration of the Fabrication Process and Thin-Film-Forming Method of an Antimicrobial and Antifouling (AP/*b*%CHG)-*grafted*-*a*%DTSACL with High Self-Healing Efficiency at Room Temperature

An earlier report by Chiu’s group proposed a poly{(*n*-butyl acrylate)_0.8_-*co*-[*N*-(hydroxymethyl)acrylamide]_0.2_} copolymer (AP) with a number-average molecular weight of 60 000 g mol^–1^, endowing high transparency as well as tunable mechanical, room-temperature self-healable, and humid-barrier properties, which was expected to be suitable as a host matrix for antimicrobial and antifouling coating applications.^38−40^ To study the influences of antimicrobial agents, we exploited a series of various ratios of DTSACL and CHG covalently and noncovalently linked to the proposed AP via a thermal-triggered cross-linking reaction (Scheme 1). The benefit of the synergistic effect based on grafting to and multiple cross-linking functions between AP segments and antimicrobial agents is to be noted. Endowed with the reactive trimethoxysilane group, DTSACL is capable of reacting with itself, leading to the formation of a polymer and simultaneously capable of covalently grafting *N*-(hydroxymethyl)acrylamide (NMA) in the AP side chain (AP-*grafted*-*a*%DTSACL), resulting in the enhancement of thermal stability, hydrophobicity, and mechanical strength. Our group discovered that the use of only DTSACL as an antimicrobial is insufficient; thus, the addition of CHG ((AP/*b*%CHG)-*grafted*-*a*%DTSACL) could improve the antimicrobial effectiveness. Through a long-term antimicrobial activity test on the (AP/*b*%CHG)-*grafted*-*a*%DTSACL thin film, we found superior antimicrobial and antifouling function (100%) against *E. coli* and *S. aureus*, which can be achieved only by adding a very tiny amount of CHG (1 wt %). Moreover, the multiple reversible hydrogen-bonding interactions from two biguanide groups of CHG generate the improvement of the self-healing function. Thus, the ease of manipulating the mechanical properties, the high transparency, the remarkable self-healing ability at room temperature, and the enhanced humidity barrier moiety of our proposed polymer demonstrate its great potential for further expansion of antimicrobial coating applications. Understanding the fascinating interaction mechanisms that occur between the three proposed compounds is crucial to enhancing their multicapabilities; hence, a series of characterizations, such as nuclear magnetic resonance (NMR), Fourier transform infrared (FTIR) spectroscopy, thermogravimetric analysis (TGA), differential scanning calorimetry (DSC), and tensile properties, were conducted.

## Results and Discussion

As illustrated in Scheme 1 (*see* the Experimental Section for details), the blended poly{(*n*-butyl acrylate)_0.8_-*co*-[*N*-(hydroxymethyl)acrylamide]_0.2_} copolymer (AP) with various concentrations of DTSACL and/or CHG was subjected to a heating process in the solid state to generate the (AP/*b*%CHG)-*grafted*-*a*%DTSACL film with many excellent features such as antimicrobial, antifouling, mechanically tunable, and room-temperature self-healing capabilities. The chemical structure of the polymer embedded with antimicrobial agents was analyzed by ^1^H nuclear magnetic resonance (^1^H NMR) spectroscopy. As discussed in previous studies, the signals at 3.98 ppm (representing the—C**H**_**2**_ group of BA) and 4.46 ppm (representing the—C**H**_**2**_ group of NMA) confirmed the successful synthesis of PBA_*x*_-*co*-PNMA_*y*_, with a desirable ratio between BA and NMA segments of 0.8:0.2. Figure S1 revealed a series of new characteristic peaks at 0.89, 1.25, 1.66, 1.77, and 3.22 ppm corresponding to −C**H**_**3**_, −C**H**_**2**_, −C**H**_**2**_–CH_2_–Si, C**H**_**2**_–CH_2_–N(CH_3_)_2_, and O–C**H**_**3**_ groups, respectively, indicating the presence of the DTSACL compound. The observation of four aromatic protons was clearly detected in the peak range of 6.55–7.73 ppm, implying the existence of CHG in the modified polymer system.^20,41^ Meanwhile, three peaks at 1.35, 1.54, and 3.04 ppm representing 12 protons in the hexamethylene chain tend to overlap with the methylene group of the other two compounds.

The reported multifunctional AP is capable of forming various interactions by a modest heating process under solution or even under the solid state owing to the functional groups from the NMA side chain.^42,43^ Taking this into account, the investigation of the effect of the antimicrobial agents (DTSACL and CHG) in AP under specified quantities was started from the physical properties. Thermogravimetric analysis (TGA) and the corresponding differential thermogravimetric analysis (DTGA) curves shown in Figure S2a–d represent the thermal degradation behavior of the cross-linked AP and various modified polymers, namely, AP-*grafted*-1%DTSACL, AP/1%CHG, and (AP/1%CHG)-*grafted*-3%DTSACL. The combination of TGA and DTGA curves facilitates the determination of maximum temperature peaks for each degradation step and simultaneously assists in the identification of degradation elements in the host compounds. As shown in Figure S2a, the thermal degradation of AP was divided into three degradation stages. The first stage is a shoulder peak with a minor weight loss of <5% at the temperature range of 100–220 °C corresponding to the degradation of functional groups and the covalent bonds formed between NMA side chains. The second stage with a drastic weight loss of around 85% at the temperature range of 220–430 °C was attributed to the cleavage of long carbon bonds in the main chains. The final stages range from 430 to 700 °C with a minor weight loss of around 10%, attributed to the carbonization of the remaining carbon chain in the polymer backbone. Meanwhile, a new peak in the temperature range of 190–300 °C is observed for all of the blended compound films, demonstrating an additional degradation step of DTSACL and/or CHG (Figure S2b–d). The new peak position was confirmed to be located in a typical degradation pattern for alkane-substituted quaternary ammonium salts and/or biguanide groups.^44−46^ The addition of 1% DTSACL resulted in a slight increase in the thermal stability of the AP from 390.3 to 392.2 °C, which is consistent with the high thermal resistance property of the silane group (Figure S2b).^47^ Meanwhile, a slight decrease in the thermal degradation of AP_1%CHG (386.4 °C) compared to AP is also suspected from the typical nature of CHG, which contains numerous degradable functional groups.

### Optimized Mechanical, Self-Recovery, and Self-Healing Properties of the (AP/*b*%CHG)-*grafted*-*a*%DTSACL Based on the Synergistic Effects of Hydrogen-Bond Interactions

**Figure 1:**
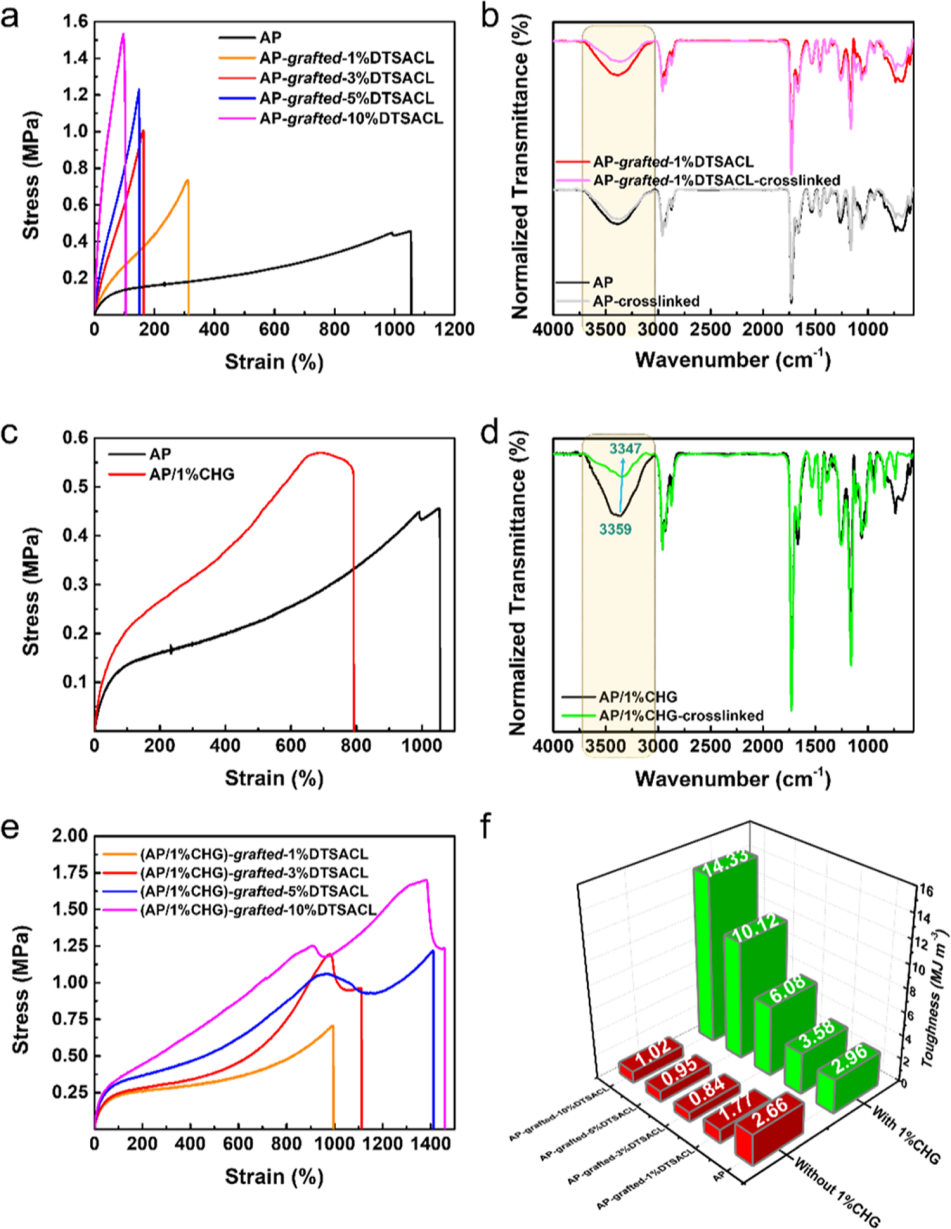
DTSACL and CHG compound-induced mechanical enhancement in the AP system. Typical stress–strain curves of (a) AP-*grafted*-1%DTSACL, (c) comparison of AP with/without the addition of 1%CHG and (e) (AP/1%CHG)-*grafted*-*a*%DTSACL. Corresponding ATR-FTIR spectra of (b) AP and AP-*grafted*-1%DTSACL and (d) AP/1%CHG before (standard) and after the heating process for 3 h, normalized with the −CH_3_ peak at 2959 cm^–1^. (f) Comparison based on the toughness values of AP and AP-*grafted*-*a*%DTSACL before and after the addition of 1%CHG. Under these experimental conditions, the contribution of antimicrobial agents in mechanical properties was basically observed.

To understand the influence of DTSACL on the mechanical properties of AP, four sets of variant wt %-DTSACL contents were added into the AP and followed by a modest heating process to form a thin film. Figure 1a displays the stress–strain curves of 0.5 mm-thick AP before and after “grafting to*”* with *a*%DTSACL (where *a*% = 1, 3, 5, and 10 wt %). Only 1 wt % DTSCL could greatly affect the mechanical behavior of AP; initially able to stretch before fracture at 1055%, it decreased drastically to 310% and continued to decrease slightly as the DTSACL concentrations were increased, resulting in a maximum strain limited to 103%. Meanwhile, the maximum strength value gradually increased from 0.46 to 1.54 MPa with the addition of DTSACL into the AP system. This mechanical enhancement can be possibly attributed to the covalently cross-linked network structure between the reactive hydroxyl group (−OH) in the NMA side chain and the trimethoxysilane group (R–O(CH_3_)_3_) in DTSACL. As shown in Figure S3, the thermally triggered covalent-bond mechanism undergoes two key reactions, hydrolysis and condensation. During exposure to a high temperature of 100 °C for 3 h, the R–O(CH_3_)_3_ group was hydrolyzed and became silanols (Si–OH). Then, the silanol group reacts covalently with the OH in the polymer chain through a condensation reaction to form a C–O–C covalent bond. Intriguingly, given that the popular silanol moieties are very reactive and can easily react under high temperatures, such a covalent cross-linked network of Si–O–Si is likely to form through a condensation-type reaction between silanols.^48,49^ The DTSACL involvement in the covalent formation degree of the AP system was explored quantitatively through attenuated total reflection-Fourier transform infrared (ATR-FTIR) spectra, as presented in Figure 1b. Through calculations by Origin software, changes in the integral area of the −OH and −NH groups (3020–3710 cm^–1^) from samples before and after being subjected to the heating process were successfully monitored. In this case, the formation of covalent bonds during the heating process is the result of the cross-linking reaction between the NMA side chain copolymer and the grafting reaction between the NMA side chain copolymer with the DTSACL. The successful formation of covalent bonds was indicated by the disappearance of the −OH and −NH reactive groups in the FTIR spectrum plot. Evidently, the marked integral area of AP-*grafted*-1%DTSACL after heating was obviously smaller compared with cross-linked AP, indicating that more covalent bonding sites were formed in AP-*grafted*-1%DTSACL. The covalent formation degree of AP-*grafted*-1%DTSACL was found to be 0.40, higher than AP (0.17). Note that the classification of the area intensity between the −OH and −NH groups is not necessary for the covalent formation degree function given that the broad peak purely represents the reactive groups.

The limitation of the tensile strain caused by the DTSACL content in the AP system could be further controlled by incorporating CHG, which has a plasticizer characteristic. We started observing the synergistic effect by comparing the results of the mechanical tensile strain on AP and AP/1%CHG. Figure 1c shows the improvement in the mechanical strength of AP/1%CHG (0.57 MPa) compared to AP and attains a splendid stretching capability of 791%. Benefitting from the two CHG biguanide groups, we suspect that the interactions between these two compounds under a thermal-triggered cross-linking reaction consist of covalent and noncovalent interactions. As shown in Figure 1d, the resultant ATR-FTIR analysis denotes strong evidence of noncovalent dynamic hydrogen-bond interactions, as indicated by a blue shift in the broad peaks associated with the overlapping −OH and −NH bands decreasing from 3359 to 3347 cm^–1^ for the uncross-linked and cross-linked AP/1%CHG, respectively. The formation of covalent bonds was confirmed by a drastic reduction in the integral area of 3020–3710 cm^–1^, implying the breakage of the −OH and −NH peaks between the NMA side chains, and between the NMA side chains and the =NH groups of CHG, followed by the enhancement of the integral area at peaks of 1256 and 1160 cm^–1^ associated with C–N and C–O–C bond formation, respectively (see the Supporting Information for details, Figure S4a,b). The cross-linking degree of AP/1%CHG was 0.69, indicating a well-constructed elaborate network structure.

To better understand the pivotal role of CHG addition toward mechanical behavior, 1 wt % CHG was added into AP-*grafted*-1%DTSACL and continued with the cross-linking process to form (AP/1%CHG)-*grafted*-*a*%DTSACL films. Remarkably, the addition of only 1 wt % CHG in AP resulted in a maximum strain greater than 3 times or more compared to AP-*grafted*-1%DTSACL, indicating that the plasticizer properties of CHG maintained good extensibility. In detail, plasticizers could improve the mechanical strain properties of the polymer by reducing the number of binding sites, as confirmed by the calculated results of the cross-linking degree of the representative (AP/1%CHG)-*grafted*-1%DTSACL reduced to 0.20 (Figure S5). The cross-linking degree of (AP/1%CHG)-*grafted*-1%DTSACL is still higher than AP, consistent with the results of the higher mechanical strength compared to AP. Even the maximum mechanical strength of (AP/1%CHG)-*grafted*-*a*%DTSACL can still compete with the AP-*grafted*-*a*%DTSACL. Close examination of Figures 1e and S6a–c revealed that the stress–strain curves (AP/1%CHG)-*grafted*-*a*%DTSACL in which the discussed DTSACL contents were 3, 5, and 10% exhibited tear-resistance properties before true fracture. As the DTSACL content increases, the notches of each sample could bear tensile strain up to 130, 450, and 553%, respectively. Besides the involvement of their innate properties, the favorable high tear resistance of the sample could be attributed to the formation of intricate structures that would cause some folded chains in the network when enough cross-linking sites between AP, DTSACL, and CHG are formed. Moreover, all modified polymers containing 1 wt % CHG showed toughness values in the range of 2.96–14.33 MJ m^–3^, much higher than those without CHG (0.84–2.66 MJ m^–3^), as summarized in Figure 1f. Of particular interest, while it is believed that the formation of complex interactions between DTSACL itself, DTSACL and AP, or CHG and AP is highly likely, interactions between DTSACL and CHG cannot be ruled out either. ^1^H NMR spectroscopy was further used to evaluate the changes in the chemical shift among the DTSACL/CHG (1:1 v/v) solution mixture before and after being heated under closed conditions at 100 °C for 3 h (Figure S7). The chemical shift to higher frequencies in the ^1^H NMR spectrum of DTSACL/CHG after heating in the CHG amide proton from 9.61 to 9.66 ppm can be attributed to the shortening of the donor–acceptor H-bond distance, which is interpreted as a physical interaction in the DTSACL/CHG mixture system (Figure S7a).^50^ Meanwhile, significant changes in the chemical shift, decrease in the signal, and the appearance of a new peak in the *para*-chlorophenyl proton at peak 6.80–7.80 (Figure S7b) indicate a tendency for CHG to form aggregates in solution.^22^ The decrease in the integral value from 0.01337 to 0.0123 in the peak range of 3.31–3.34 ppm belonging to the methoxy proton DTSACL (Figure S7c) confirms the conversion of −OCH_3_ into covalent bonds, forming C–O–C. The structural complexity of these randomly modified compounds can be advantageous for overcoming the trade-off between self-healing and good mechanical compliance.

In view of advanced applications such as skin-like wearable electronic sensors and display systems,^51−53^ emerging antimicrobial materials with mechanical properties similar to human skin are highly favorable to withstand various deformation forces due to complex motion. The proposed compound is recommended to be able to tolerate various deformations up to the range of 140–180% with elastic moduli of 0.5–1.95 MPa.^43^ Thus, repeated loading–unloading cycle tensile tests for each compound were carried out with increasing maximum strain from 40 to 200% and within a short rest period for each loading cycle of 5 min. Based on the cyclic test results among AP, AP-*grafted*-1%DTSACL, and AP-*grafted*-3%DTSACL shown in Figure S8a–c, it was found that the hysteresis area value decreased drastically as the DTSACL content in the AP system increased, indicating that the synergistic effects based on covalent and noncovalent interactions between two compounds provided more powerful energy dissipation compared to pristine AP. The formation of covalent bonds serves an important role in strengthening and maintaining the polymer network. Meanwhile, the reversible H-bonds can dynamically cleave and reform during the loading–unloading tests. Figure S8d,e shows the limitation of the stretching ability of the modified compounds (not up to 100%) as a result of the excess covalent-bond formation leading to stiffness. Figure S8f–j shows the effect of adding 1% CHG on the AP-*grafted*-*a*%DTSACL elasticity. Owing to the plasticizer characteristic from CHG and the rigidity property from DTSACL, the hysteresis area value increased. Interestingly, all compounds showed satisfactory self-recoverability, which was able to return to their original size by only 5 min of rest. The hysteresis ratio of AP-*grafted*-3%DTSACL is the lowest in the ten samples, which is ascribed to the perfect repeat cycle behavior. The profitable cyclic mechanical properties of these modified compounds rendered them potential antimicrobial protector candidates for use in external wearable electronic devices.

Knowing the compatibility of the host polymers is a crucial prerequisite in evaluating their potential use in a variety of applications that rely on direct contact with human skin. Our group further summarized the compatibility of the poly(butyl acrylate) (PBA) and poly(*N*-hydroxymethyl acrylamide) (PNMA) used for this research (Table S2). Through in vitro cytotoxicity tests on L929 cells, it was reported that PBA was nontoxic.^54^ Owing to the possibility of modifications in BA units of the polymer chain that can impart new properties in the polymers, it was found that modified PBA has enormous importance in the medicinal and biological areas.^55^ For example, Lendlein et al. introduced soft covalently cross-linked PBA networks (cPnBA) as sterilizable, nontoxic, and immuno-compatible biomaterials with mechanical properties adjustable to human vascular smooth muscle cells and aortic fibroblasts.^55^ To further expand the mechanically dependent applications, the copolymers of butyl acrylate with hydroxyl ethyl methacrylate (HEMA), propylene glycol dimethacrylate (PGDMA), and methyl methacrylate (MMA) networks have been proposed and reported to be biocompatible as evaluated by in vitro cytotoxicity tests (Table S2).^54−56^ Meanwhile, NMA as a hard segment that includes two hydrophilic groups of amide and hydroxyl separated by a methylene group was often copolymerized with other monomers to produce nontoxic and biocompatible copolymers for applications in wearable sensors, drug loading, and in various fields of biotechnology (Table S2).^57−59^ Relying on the good biocompatibility, polymers composed of BA and NMA monomers are potentially safe for direct use on human skin.

**Figure 2:**
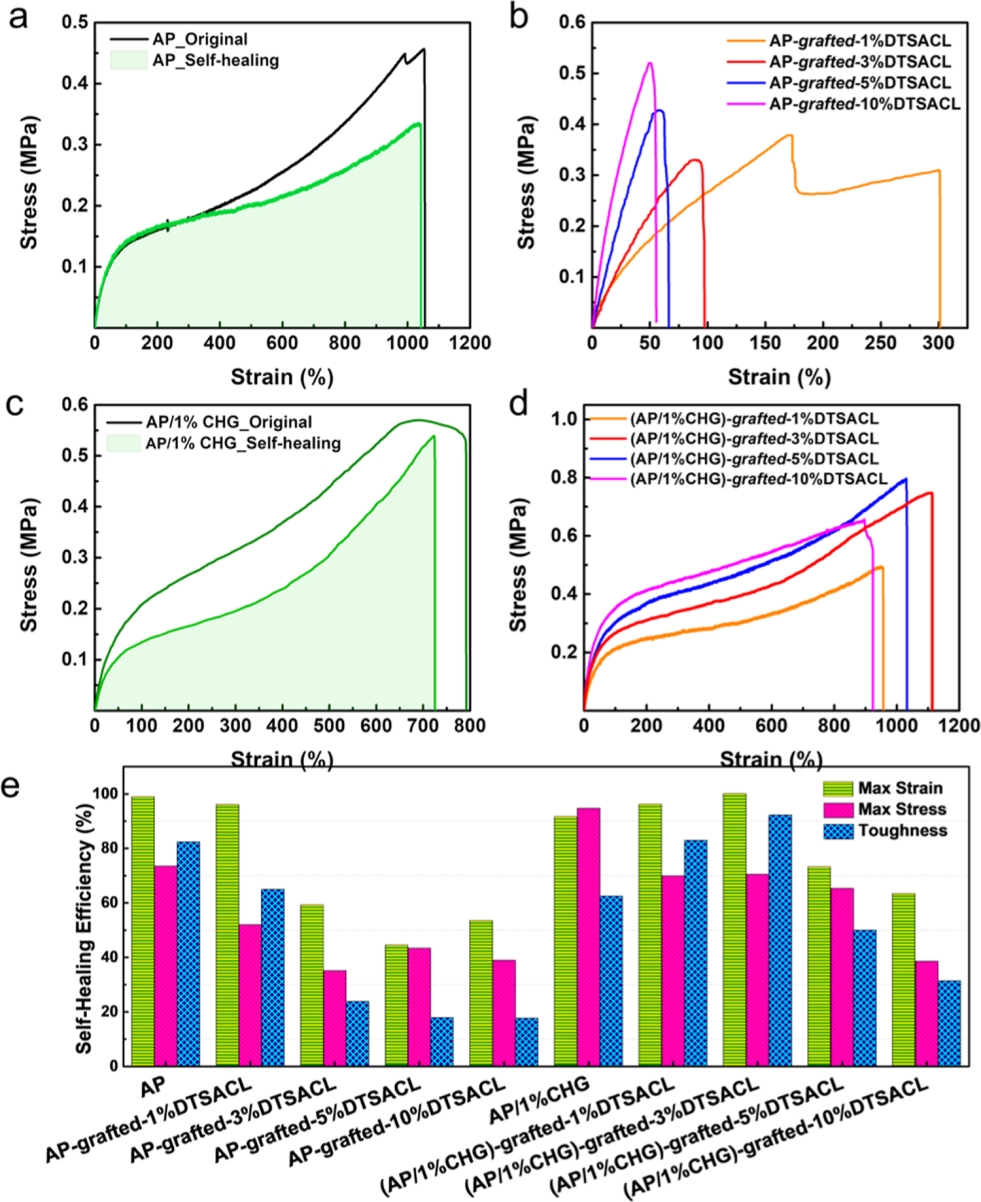
Investigation of the self-healing capabilities quantitatively through tensile measurements focusing on (a) original and self-healed AP samples; (b) self-healed AP-*grafted*-*a*%DTSACL; (c) original and self-healed AP/1%CHG; (d) self-healed (AP/1%CHG)-*grafted*-*a*%DTSACL; and (e) the corresponding self-healing efficiencies based on the proportion of recovered strain–stress-toughness with respect to the initial strain–stress-toughness values.

Soft self-healing materials based on the incorporation of weak and reversible noncovalent interactions (i.e., hydrogen bonds, ionic bonds, and metal–ligand interactions) into polymer networks have become a popular strategy frequently proposed by researchers.^60−63^ A similar strategy was also employed by our group. To probe the full-cut self-healing properties, nine types of rectangular virgin samples were completely cut into half by a feather disposable scalpel No. 3 with accommodate blade No. 10 carbon steel and then immediately reconnected at both damaged ends for 24 h at room temperature without external stimuli. Each healed sample was further subjected to a tensile test. Thanks to the dynamic nature of hydrogen bonds on the NMA segment and the high chain mobility facilitated by the BA segment, the mechanical properties of the healed AP film approached the original values of the AP film (Figure 2a). The healing on the mechanical properties was quantitatively determined as 99, 74, and 82% based on the maximum strain, maximum stress, and toughness, respectively. Meanwhile, the four AP-*grafted*-*a*%DTSACL samples seem to display a decrease in the self-healing ability along with an increased DTSACL loading (Figure 2b). These are obviously due to the formation of covalent bonds from the trimethoxysilane groups of DTSACL, with the NMA side chain suppressing the molecular chain movement in the copolymer system, thus hindering dynamic hydrogen-bond interactions. In contrast to the influence of DTSACL loading, CHG possesses plasticizer characteristics and provides numerous—NH groups from two biguanide structures that can easily interact with the NMA side chain, leading to the improvement of the self-healing capability based on weak and reversible noncovalent interactions via hydrogen bonds. Thus, the presence of only 1 wt % CHG in the AP (Figure 2c) and in the AP-*grafted*-*a*%DTSACL (Figure 2d) improved the self-healing ability. All healed films containing 1 wt % CHG could be easily stretched over 700% strain after being healed for 24 h. A column chart in Figure 2e demonstrates that the AP, AP-*grafted*-*a*%DTSACL, AP/1%CHG, and (AP/1%CHG)-*grafted*-3%DTSACL samples possess the best self-healing efficiencies and even exhibited very competitive room-temperature self-healing capabilities when compared to other reported ones.^16,28−33,35^ As the self-healing polymer concept adopted in this work is based on chain movement diffusion for the spontaneous rearrangement of reversible dynamic hydrogen bonds, measurement of the glass-transition temperature (*T*_g_) by differential scanning calorimetry (DSC) is required. Figure S9 shows that the *T*_g_ values of all of the representative cross-linked samples such as AP, AP-*grafted*-*a*%DTSACL, AP/1%CHG, and (AP/1%CHG)-*grafted*-3%DTSACL were not higher than −12 °C, confirming such compounds are capable of facilitating room-temperature self-healing ability.

### Self-Healing Ability of Transparent AP and Modified Polymers in Microthick Films

**Figure 3:**
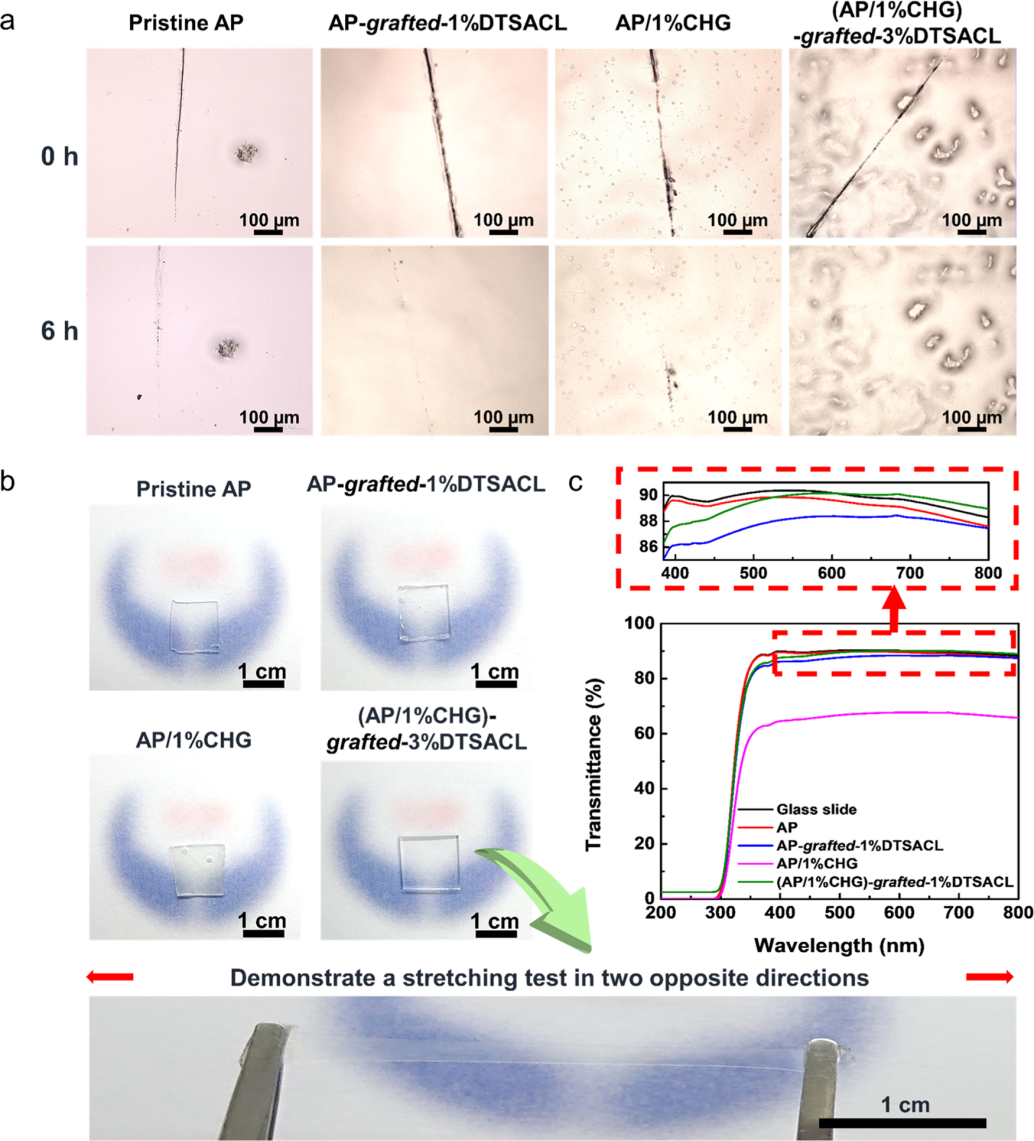
(a) Visual observation of the self-healing process at room temperature for 6 h of AP, AP-*grafted*-1%DTSACL, AP/1%CHG, and AP/1%CHG-*grafted*-3%DTSACL via an optical microscope (OM) (scale bar = 100 μm). The average scratch widths for AP, AP-*grafted*-1%DTSACL, AP/1%CHG, and (AP/1%CHG)-*grafted*-3%DTSACL were 7.90 ± 1.79, 19.80 ± 5.69, 10.80 ± 3.97, and 12.90 ± 6.01 μm, respectively. (b) Photographs of pristine AP and modified polymers with different types and concentrations of antimicrobial agents in the form of a thin film with a thickness of 0.5 mm, showing impressive transparency. Demonstration of (AP/1%CHG)-*grafted*-3%DTSACL sustains a large stretching capability (scale bar = 1 cm). (c) Measurement transparency results of microscope slide glass and microscope slide glass coated with AP and modified polymers via a UV–vis spectrometer.

A series of the prepared thin-film samples such as AP, AP-*grafted*-1%DTSACL, AP/1%CHG, and (AP/1%CHG)-*grafted*-3%DTSACL with thicknesses of 2.70 ± 0.64, 4.04 ± 0.68, 3.10 ± 1.68, and 4.44 ± 1.24 μm, respectively, were subjected to a scratch recovery test and further traced by an optical microscope (OM). Considering that the scratches in the materials were created manually using a feather disposable scalpel, there can be a possibility of the width varying throughout the whole scratch line. Hence, the scratch width was calculated by averaging ten different locations along the scratch line, resulting in 7.90 ± 1.79, 19.80 ± 5.69, 10.80 ± 3.97, and 12.90 ± 6.01 μm for AP, AP-*grafted*-1%DTSACL, AP/1%CHG, and (AP/1%CHG)-*grafted*-3%DTSACL, respectively. It can be observed that the scratches made by the feather disposable scalpel on all sample surfaces became narrow and completely healed within 6 h at room temperature (Figure 3a), especially on AP/1%CHG and (AP/1%CHG)-*grafted*-3%DTSACL, which completely healed without leaving marks like the other two films, implying that the presence of a small amount of CHG in the AP system promoted self-healing beyond molecular chain diffusion.

Simultaneously, OM was also used to observe the morphology of polymeric surface thin films with and without an admixture of DTSACL and/or CHG. It can be seen that the addition of 1% DTSACL into the AP does not have a significant effect on the surface, which remains smooth, suggesting the good compatibility between hydrophobic DTSACL and AP. The surface of the AP/1%CHG film appeared to be relatively smooth, indicating that CHG was well-incorporated into the films with small spherical particles that could be due to CHG precipitates generated during solvent evaporation. Quantitative insights into the elemental composition of CHG embedded into the AP were further analyzed by using X-ray photoelectron spectroscopy (XPS). The acquired full survey spectra and the corresponding element area, surface atomic compositions, and functionality ratios of AP and AP/1%CHG are given in Figure S10a,b and Table S3, respectively. It is apparent that the N percentage increases after the incorporation of CHG into the polymer due to the contribution of two biguanide groups. Moreover, the existence of Cl element from CHG is also detected in the AP/1%CHG, suggesting that CHG has been successfully embedded into the AP. To thoroughly observe the uniform distribution of CHG in the AP/1%CHG thin film, energy-dispersive X-ray (EDX) spectroscopy was employed to map the element Cl distribution, which is indicative of CHG because the polymer host only contains C, N, and O elements as previously verified by XPS analysis (Figure S10a,b). As outlined in Figure S11a,b, the resulting field emission scanning electron microscopy (FE-SEM) image and elemental mapping manifest the uniform distribution of CHG within a polymeric network. The EDX spectrum provides detailed information regarding the weight percentages of the elements C, N, O, and Cl as 56.37, 18.68, 23.91, and 1.05%, respectively (Figure S10c). Meanwhile, incorporating 3% DTSACL and 1% CHG into the AP system resulted in a rough surface with an observable irregular and large particle-like shape. This is very likely due to the tendency of DTSACL to interact first with the precipitated CHG. Supported by the increased presence of DTSACL in adjacent chains, more inter- and intramolecular covalent and noncovalent bonds are easily formed in the cross-linked polymer system when the grafting content of DTSACL reaches 3%. Such complex covalent and noncovalent bonds cause aggregate formation in the matrix. This result is consistent with the ^1^H NMR analysis result (Figure S7a–c).

Given the antimicrobial function is widely applied in various fields, transparency is one of the crucial features, especially as an antimicrobial screen protector on optical and display devices.^16,64,65^Figure 3b shows that all of the highly stretchable proposed compounds consisting of AP, AP-*grafted*-3%DTSACL, AP/1%CHG, and (AP/1%CHG)-*grafted*-3%DTSACL with a thickness of 0.5 mm were colorless and displayed excellent transparency as evidenced by the underneath images of the modified polymers that can be observed clearly, except for AP/1%CHG, which was slightly cloudy. Nonetheless, the addition of DTSACL (3 wt %) in this work allowed for a neutral transparent material (Figure 3b). In practice, sample coatings are usually applied onto films as thin as a few micrometers thick. Thus, the high optical transparency of the blended compounds coating onto a microscope slide glass was further evaluated quantitatively via a UV–vis spectrometer (Figure 3c). The transmission of all blended compounds except for AP/1%CHG exceeds 86% (close to the glass) at around 395 nm, suggesting that the films are transparent starting from the UVA and UVV wavelengths (320–400 nm) to the visible red region (620–750 nm). Importantly, the (AP/1%CHG)-*grafted*-3%DTSACL film displays slightly better transparency compared to the pure AP film in the wavelength range of 540–800 nm, even higher than the value of bare glass starting from 620 to 800 nm wavelength, as shown in the zoomed-in image in Figure 3c. This influence is attributed to the lower refractive index of DTSACL than AP and glass at certain wavelengths, attributed to the fact that more light can pass through the surface.^66^ The transparency of glass coated with AP/1%CHG can still be maintained in the limit of 67%. Such results were reasonable, as the transparency value certainly increases with the thinning of the blended compound films. The exceptionally high transparency of the prepared coatings indicates that the proposed modified compounds can compete with other transparent elastomers.^16,66,67^

### Effect of DTSACL and CHG on the Hydrophobic Properties of Modified Polymer Films

**Figure 4:**
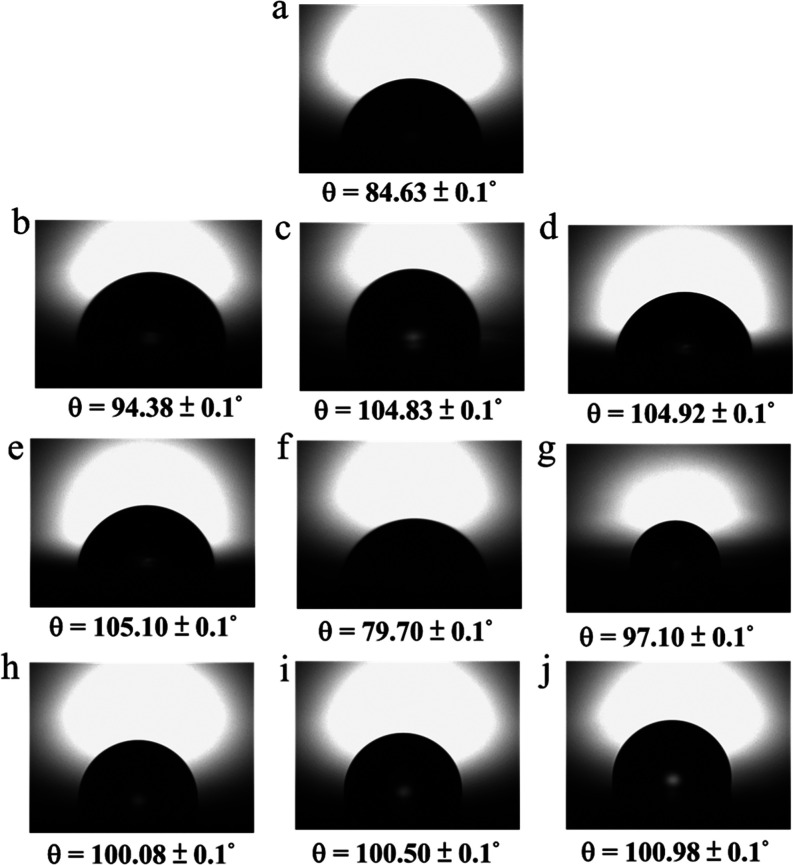
Images of the water contact angles (WCA) test results of (a) AP, (b) AP-*grafted*-1%DTSACL, (c) AP-*grafted*-3%DTSACL, (d) AP-*grafted*-5%DTSACL, (e) AP-*grafted*-10%DTSACL, (f) AP/1%CHG, (g) (AP/1%CHG)-*grafted*-1%DTSACL, (h) (AP/1%CHG)-*grafted*-3%DTSACL, (i) (AP/1%CHG)-*grafted*-5%DTSACL, and (j) (AP/1%CHG)-*grafted*-10%DTSACL thin-film coating on the silica wafer surface followed by a cross-linking reaction for 3 h at 100 °C. Representation of the improvement in hydrophobicity as the DTSACL content increases in AP systems and vice versa with the incorporation of CHG.

Although the antimicrobial film is mostly designed with hydrophilic surfaces, the development of an antimicrobial film with hydrophobic surfaces is also needed to provide water-resistant properties, particularly for precious instruments, electronic devices, umbrellas, waterproof building materials, etc.^13,67^Figure 4a–j summarizes the results of the water contact angle (WCA) measurements on silica wafer substrates with and without coating by the different concentrations of (AP/1%CHG)-*grafted*-*a*%DTSACL. The WCA value of the cross-linked AP was 84.63° (Figure 4a) and tended to increase toward hydrophobicity as the DTSACL content was increased (Figure 4b–e). The retention of increased hydrophobicity is most likely due to the presence of the aliphatic octadecyl chain and enhancement of the covalent bonds during the “grafting to” reaction in the modified polymer system (Figure 1b). In contrast, the presence of CHG tends to increase the surface hydrophilic characteristics due to the presence of the polar amide from the biguanide group (as has been explained in Figures 1d and S4a,b). Figure 4f shows that the WCA measurement result of AP/1%CHG decreased to 79.7°. Interestingly, the combination of DTSACL and CHG in the AP system still maintains surface hydrophobicity above 97° (Figure 1g–j).

### Antimicrobial Evaluations

In order to be categorized for proper repeated use in real life, especially for applications that are in direct contact with humans such as electronic skin, the safety and cleanliness of electronic devices must be considered. Thus, electronic devices that are coated with effective antimicrobial compounds are needed to avoid infection during the use process. Two common germs that often cause infectious diseases in our daily lives are Gram-negative (*E. coli*) and Gram-positive species (*S. aureus*), which were selected as the model bacteria to evaluate the bactericidal ability of the sample. The killing rates against *E. coli* and *S. aureus* by the pristine polymer and the modified polymer were further determined by the AATCC method. Figure S12 presents the bacteria colonies on AP and the modified polymer after being cocultured with *E. coli*. The pristine AP possessed a killing rate of about 76% against *E. coli*. Overall, it takes 5% DTSACL to completely kill *E. coli*, while only 1% CHG is required to eliminate all of the bacteria. Figure S13 shows that the bactericidal ability of the blended compounds against *S. aureus* exhibited the bacteria killing rate reaching 100% by only employing AP/1%CHG, much better than using AP-*grafted*-10%DTSACL, which still showed plenty of viable bacteria on the surface. This demonstrates that CHG in AP is more efficient as an antibacterial agent against various germs and is more feasible in preventing the formation of a biofilm on the surface. These results are in accordance with what has been reported,^68−70^ where the quaternary ammonium salt type has a relatively lower antibacterial efficacy as compared with N-halamine compounds due to the presence of the hydrophobic aliphatic octadecyl chain.

**Figure 5:**
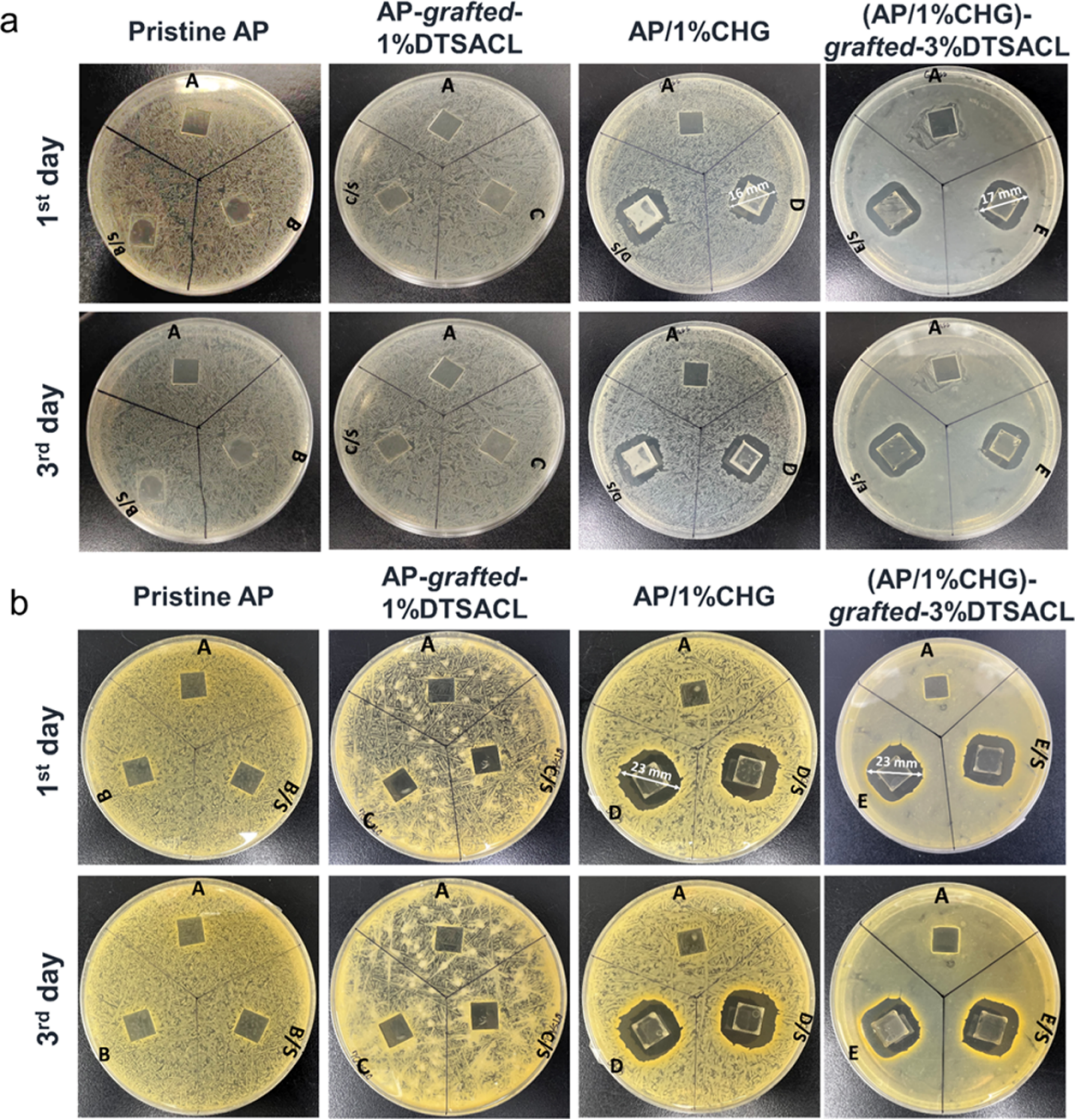
AP and modified polymers’ coating on the glass substrate were laid on 16 h grown (a) *E. coli* and (b) *S. aureus* colony lawns, and the inhibition zones were observed on the first and third days. Each Petri dish consists of [A] a pristine glass substrate, [B–E] coating the AP, AP-*grafted*-1%DTSACL, AP/1%CHG, and (AP/1%CHG)-*grafted*-3%DTSACL on the glass surface, respectively, and [B/S–E/S] the same coating film under the scratched condition.

The antibacterial activity of AP and modified polymers triggered by the growth of bacteria was further investigated by the bacterial inhibition zones. One × 1 cm^2^ rectangle glass substrate coated with a selected set of materials based on satisfaction of mechanical, self-healing, and bactericidal abilities, namely, AP, AP-*grafted*-1%DTSACL, AP/1%CHG, and (AP/1%CHG)-*grafted*-3%DTSACL, was prepared. As shown in Figure 5, each Petri dish was divided into three regions consisting of a glass substrate only as the control sample, coated glass, and coated glass under scratched conditions. As could be noticed from Figure 5a, glass coated with AP/1%CHG and (AP/1%CHG)-*grafted*-3%DTSACL is more effective in inhibiting the growth of *E. coli* bacteria than AP and AP_1%DTSACL. From the first to the third day, the inhibition zone was clearly visible and there was no change in the diagonal size of the inhibition zone, suggesting that the antimicrobial agents with positively charged compounds disrupted the negative charges on the bacterial membrane and bound to the bacterial DNA, thus effectively eliminating the existence of bacterial colonies and preventing bacteria from approaching the coated glass. The antimicrobial agent had a successful long-term function and was long enough for the self-healing process to be completed. Therefore, there was no significant difference in the inhibition zone between the unscratched and scratched film coating on the glass surface. Similar growth-triggered antimicrobial activity of blended AP/antimicrobial agents against *S. aureus* was also observed as shown in Figure 5b. The diagonal size of the inhibition zone of (AP/1%CHG)-*grafted*-3%DTSACL was 23 mm, confirming the favorable antimicrobial properties against *S. aureus* compared to *E. coli* (17 mm).

An important long-term antifungal efficacy of (AP/1%CHG)-*grafted*-3%DTSACL was observed visually (Figure S14). Since it is known that fungal contamination is the most common and easy method of spoilage of foods such as bread,^71^ our group conducted a preliminary test to assess the antifungal efficacy by embedding one side of the surface of a silica wafer coated with (AP/1%CHG)-*grafted*-3%DTSACL into bread and being left in a plastic wrapped condition at room temperature for 21 days. The fungi grew rapidly and almost covered the whole bread; interestingly, no fungal growth occurred near the area where the silica wafer was embedded with the select compound blend. Considering that the antimicrobial polymer also emphasizes its elasticity for potential application in an electronic skin, a shelf-life study of preserved white bread was conducted by directly allowing the (AP/1%CHG)-*grafted*-3%DTSACL solution to adsorb into the sponge-textured white bread (Figure S15a). The shelf life of the white bread treated with antimicrobial protection was significantly extended to more than 2 weeks. The absence of fungal growth in all areas of the bread and the ability to maintain the hardness of the bread texture demonstrate the high efficiency of (AP/1%CHG)-*grafted*-3% DTSACL as an antimicrobial polymer, even in humid environmental conditions for long-term use. These results also imply that the elasticity of the antimicrobial polymer allows easy coating on the entire surface of the curved and irregularly shaped white bread. Meanwhile, a slice of white bread without antimicrobial treatment as the control sample was found to be spoilt 3 days after storage (Figure S15b). These results prove its great potential in terms of antimicrobial protective coating applications.

### Environmental Impact of (AP/*b*%CHG)-*grafted*-*a*%DTSACL Coatings

The ideal antimicrobial coating must be nontoxic while effectively maintaining efficacy over a long period of time, which is in accordance with the advantages of DTSACL and CHG possessing relatively less toxicity and can be used in contact with human skin.^19−22,49^ What needs to be considered now is their stability in the scope of human habitation, under warm (27–35 °C) and humid environments. This section focuses on the advantages of existing (AP/*b*%CHG)-*grafted*-*a*%DTSACL properties with their impact on the environment. The main value proposition of this research topic relies on leveraging both covalent and noncovalent interaction complexes triggered by thermal cross-linking reactions to control the instability arising from the hydrophilic properties originating from NMA and CHG under humid conditions. The dominance of BA and DTSACL along with thermal cross-linking reactions can improve the hydrophobic property, thereby reducing the susceptibility of the proposed compound to humidity.

As part of our experimental validation, we conducted water contact angle measurements of pristine AP and AP embedded with CHG or DTSACL before and after the cross-linking reaction to assess the impact of the resulting covalent bonds on the hydrophobicity of the material. This approach allowed us to directly observe and quantify any changes in the material’s surface properties, specifically its ability to repel water, thereby providing empirical evidence of the effectiveness of the thermal cross-linking process in enhancing hydrophobic characteristics. Based on Figure S16, it is reported that measuring contact angles on the copolymer surfaces of AP, AP-*grafted*-1%DTSACL, AP/1%CHG, and (AP/1%CHG)-*grafted*-3%DTSACL before and after 3 h of cross-linking at 100 °C can concretely confirm the stability of hydrophobicity, as validated by the increase in WCA after cross-linking. Meanwhile, there is no issue with resistance to high temperatures considering that the pristine polymer and the polymer embedded with CHG and/or DTSACL degradation temperature (Td^5%^) were above 200 °C (Figure S2).

The introduction of the self-healing ability of the proposed polymer can greatly extend the lifecycle. Thus, we minimize the need for frequent replacement and associated resource consumption. Overall, by providing self-healing capabilities, stability to high temperatures, and reduced susceptibility to humidity, the proposed self-healing coating material aids in reducing waste generation through preventing early disposal.

### Prospective Large-Scale Production of the Self-Healable Cross-Linkable (AP/*b*%CHG)-*grafted*-*a*%DTSACL as an Antimicrobial Surface Coating

First of all, we emphasize that a proof-of-principle of fabricating a copolymer with multiple functionalities is feasible to scale up through free radical polymerization (FRP). Owing to the possibility of converting a wide variety of vinyl monomers (sometimes possessing functional groups) into high-molecular-weight polymers without rigorous purification of the monomers and solvents, FRP is considered to be one of the most feasible methods not only for the laboratory but also for industrial scale processes. In this work, polymerization was carried out for 24 h at 65 °C, resulting in a high yield of 90% with a number-average molecular weight Mn_(GPC)_ of 60 000 g mol^–1^ and a PDI value of 1.19.^39^ It should be noted that the choice of reaction time of 24 h was based on the certainty that the reaction would reach saturation conditions. For effectiveness and cost savings in the production process, further investigation of kinetic polymerization is necessary. However, the procedure for incorporating polymers with antimicrobial agents at the specified wt % variation was through a simple blending process, solvent elimination at room temperature, and ending with a thermal-triggered cross-linking reaction at 100 °C for 3 h.

The second consideration is that all of the chemicals proposed for the synthesis of the (AP/*b*%CHG)-*grafted*-*a*%DTSACL film were commercially available at affordable prices (Table S4). As a comparison study, our group focused on hydrophobins as one of the antimicrobial substances. Hydrophobins are a type of protein produced exclusively by filamentous fungi that are considered to be the most surface-active class of cysteine-rich proteins.^72,73^ They are capable of spontaneously assembling into amphipathic monolayers at hydrophobic–hydrophilic interfaces that alter the surface wettability (turning a hydrophobic surface into a hydrophilic surface and vice versa) and possess a specific property of producing stable foam. The potential biotechnological applications of hydrophobins rely on their ability to reverse the hydrophilic–hydrophobic character of a surface or their surfactant capacity. Current popular research uses the hydrophobin Schizophyllum commune (SC3) to coat nitric oxide (NO) releasing medical-grade polymers to establish an antifouling layer to work synergistically with the bactericidal and antiplatelet activities of NO (SC3-NO).^73^ However, large-scale hydrophobin production might be difficult to implement due to the complexity of the manufacturing and isolation process, the production cost of recombinant proteins, and/or the large-scale requirements of the proteins.^72^ A real example of the difficulty in manufacturing hydrophobins in large-scale production can be seen from the sales for 1 mL of hydrophobin SC3 according to Sigma-Aldrich (CAS: 68795), which is 1709 USD. To this extent, we humbly claim that the cost of required chemicals in our platform is not really a significant issue compared to hydrophobin SC3 as the existing antimicrobial coating. At the same time, these results further emphasize that our developed platform for multifunctional antimicrobial materials offers many benefits, starting from cost efficiency, high production yield with various tempting properties, and the possibility of being used on various types of substrates.

Despite these potentials, there are three underlying problems in the production of (AP/*b*%CHG)-*grafted*-*a*%DTSACL. At first, when carrying out large-scale production, the differences in the equipment used need to be considered, such as the reaction process using a reactor and the process of separating polymer products from methanol as the solvent using a distillation column. Automatically, reconditioning is required such as material composition, temperature and reaction time, speed, and type of mixer. In addition, batch or continuous type of reactor operation, quality monitoring including the physical, mechanical, and chemical properties of the materials used, toxicity, and reactivity with humid air and oxygen are mandatory to be taken into consideration to ensure a low error rate. Challenges that may arise in automated manufacturing and quality control must be addressed from the design stage by following a simultaneous engineering approach. The next challenge is to integrate CHG and DTSACL as antimicrobial agents into the polymer system while considering even distribution or avoiding the possibility of easy aggregation. Therefore, further investigation into the solubility and incompatibility of the added antimicrobial components with a polymer is necessary. Lastly, the production environment needs to be considered because of its vital role in determining the manufacturing cost, coupled with the knowledge of environmental necessities for legal regulations and the ecological footprint.

## Conclusions

We have successfully prepared a series of self-healing and antimicrobial modified polymer coatings by incorporating a tiny amount of DTSACL and/or CHG into PBA_0.8_-*co*-PNMA_0.2_. The perfection of these three multifunctional compound combinations can be described as follows. (1) The antimicrobial efficacy not only helps kill bacterial cells but can also improve the mechanical properties, the autonomous self-healing ability, and self-recoverability through a synergistic effect between AP and antimicrobial compounds, which are essential for maintaining optimal performance over a long operation life span. (2) The hydrophobic moiety provides water resistance and assists in reducing the initial microbial attachment on the surface substrate. Lastly, (3) it is common for compounds to turn cloudy after CHG addition; interestingly, our group found that integration with DTSACL can maintain high transparency in the modified copolymer. Thus, the proposed material is expected to be implemented for further expansion of applications such as an antimicrobial screen protector on wearable optical and display devices. Under a proper blend concentration ratio between AP and antimicrobial agents, the resulting mechanical properties were considerably better than pristine AP, where the high toughness of the modified polymer increased 5 times compared to AP from 2.66 to 14.33 MJ m^–3^. It was found that only adding 1 wt % CHG resulted in improved tear resistance, complete healing upon scratches caused by external forces within 6 h, and even over 80% self-healing efficiency on full-cut within 24 h. We further demonstrated that the thin-film modified polymer with a microthickness coating on a glass surface could perfectly eliminate *E. coli* and *S. aureus* via a bacterial inhibition zone test. In addition, the as-prepared (AP/1%CHG)-*grafted*-3%DTSACL demonstrated long-term (21 days) antifungal properties in bread. As the synthesis method is feasible to scale up and the antimicrobial agents proposed are commercially available commodities, we expected that such a strategy and the choice of compounds used in this work could be developed as multifunctional antimicrobial screen protectors for various applications.

## Experimental Section

### Materials

Butyl acrylate (BA) was purchased from Sigma-Aldrich and was then purified by passing it through an alumina column. *N*-(Hydroxymethyl)-acrylamide (NMA) and azoisobutyronitrile (AIBN) were purchased from Tokyo Chemical Industry Co. Dimethyl octadecyl(3-trimethoxysilylpropyl) ammonium chloride (DTSACL) was obtained from Acros Organics. Chlorhexidine gluconate (CHG) was supplied by Chengyi Chemical Raw Materials Co., Ltd. and used without any further processes.

### Characterizations

The ^1^H nuclear magnetic resonance (^1^H NMR) spectra were measured on a Bruker AVANCE III HD-600 spectrometer operating at 600.17 MHz in DMSO-*d*_6_ at 20 °C, and the sample concentrations were in the range of 5–10 wt %. Ultraviolet–visible (UV–vis) absorption of the specimens was characterized with a UV–visible spectrophotometer (Jasco V676).

To investigate the synergistic effect between flexible and self-healable AP with antimicrobial agents, a series of thin-film samples consisting of pristine AP and modified polymers before and after the heating process were analyzed by attenuated total reflectance Fourier transform infrared spectroscopy (ATR-FTIR, PerkinElmer, Frontier) in the scanning range of 565–4000 cm^–1^ at 25 °C. The thin films were dried overnight under a vacuum before analysis. To eliminate any effects of the ATR crystal contacting the samples, the ATR-FTIR spectra were normalized using the C–CH_3_ peak representing the BA segment at 2959 cm^–1^, whose intensity was not changed during the cross-linking reaction. The cross-linking degree value was determined by the change in the integral area of the −OH and −NH peaks (3020–3710 cm^–1^) from samples before and after the heating process and was calculated using Origin software with a spline baseline function.

Thermal degradation analysis of thermally triggered cross-linked specimens at 100 °C for 3 h consisting of amphiphilic copolymer (AP) and polymer compounds loaded with different mass percentages of DTSACL and with definite mass percentages of CHG (1 wt %) was carried out by a thermogravimetric analyzer (TGA, 550/TA) in the temperature range of 25–700 °C at a constant heating rate of 10 °C min^–1^ under a nitrogen atmosphere with a gas flow rate of 50 mL min^–1^. Prior to measurements, all specimens were kept in a vacuum oven at 40 °C for 6 h to avoid the influence of moisture. Differential thermogravimetric analysis (DTGA) was obtained by calculating the first derivative of the available TGA data.

The differential scanning calorimetry (DSC) measurements were carried out using a DSC2-00573 instrument (192.168.0.5). A ∼5 mg dry sample was subjected to the following temperature program: (I) equilibrate for 5 min at −80 °C; (II) heat from −80 to 100 °C and hold for 5 min; (III) cool from 100 to −80 °C and hold for 5 min, which is marked as the end of the first cycle. The measurement of the glass-transition temperature (*T*_g_) for each sample was carried out in two cycles at a heating rate of 10 °C min^–1^.

Surface chemistry examination of AP and AP/1%CHG was conducted by using XPS (ULVAC-PHI (Quantes)-ESCA0038) with a monochromatic Al–Kα emitter as the source gun type. The surface morphology of AP/1%CHG was observed using FE-SEM JEOL JSM-6700F at an accelerating voltage of 0.5–30 kV, and the elemental composition present in AP/1%CHG was determined using EDX.

Mechanical, tensile cyclic, and self-healing tests under macroscale thickness were performed on a universal testing machine setup (Shimadzu EZ-EX) at a strain rate of 50 mm min^–1^ and were recorded with Trapezium X software. The dimension of the tensile test specimen with a rectangle shape was 30 mm (length) × 10 mm (width) × 0.5 mm (thickness). For the self-healing evaluation, the specimen was cut into separate halves with a feather disposable scalpel No. 3 with accommodate blade No. 10 carbon steel and then gently put back in contact. The sample films were healed for 24 h under ambient conditions with an average relative humidity value of 55 ± 5% at 25 ± 2 °C as measured by a large screen LCD digital electronic temperature and humidity meter WD-5016. The self-healing efficiencies (%) are defined as the ratio of maximum stress, maximum strain, or toughness of healed and virgin materials.

The scratch repair procedure is as follows. The surface of the microthickness sample coated on the microscope slide glass was scratched using a single-sided blade and left at room temperature to wait for the surface to repair itself within 6 h. The thickness of each sample was measured by a Microfigure Measuring Instrument-Surfcorder ET3000 2 with a force around 30–35 μN and a range of 1.65 μm at a constant speed of 50 μm s^–1^.

The influence of DTSACL and CHG addition in the AP system toward the surface hydrophobicity or hydrophilicity degree was studied by means of contact angle measurements (MCA-3 Kyowa Interface Science, Japan).

### Preparation of AP

The flexible and self-healing PBA_*x*_-*co*-PNMA_*y*_ with a desired ratio between BA and NMA segments used of 0.8:0.2 and number-average molecular weight (*M*_n_) of 60 000 g mol^–1^ was synthesized by the FRP method as described in a previous study.^39^

### Preparation of Modified Polymers (AP/*b*%CHG-*grafted*-*a*%DTSACL) in the Bulk Form

400 mg of AP and various mass percentages of DTSACL and CHG to the total polymer mass (Table 1) were blended with methanol as the solvent at room temperature for 15 min and stirred at 1000 rpm so that a homogeneous mixture could be obtained. The blended compound was poured into a Teflon mold and dried under ambient conditions for 2 days followed by a thermal-triggered cross-linking reaction for 3 h at 100 °C, yielding AP/*b*%CHG-*grafted*-*a*%DTSACL modified polymers in the solid-state form. The resulting rectangular-shaped modified polymer films with dimensions of 15 mm in length, 8 mm in width, and 0.4 mm in thickness were then used for mechanical, self-healing, and repeated loading–unloading tensile cyclic tests.

**Table 1:** Sample Code and Preparation Condition

sample composition	sample code	DTSACL (μL; mmol)	CHG (μL; mmol)
PBA_0.8_-*co*-PNMA_0.2_	AP		
PBA_0.8_-*co*-PNMA_0.2_ + 1 wt DTSACL	AP-*grafted*-1%DTSACL	33.4; 0.0358	
PBA_0.8_-*co*-PNMA_0.2_ + 3 wt % DTSACL	AP-*grafted*-3%DTSACL	100.0; 0.1076	
PBA_0.8_-*co*-PNMA_0.2_ + 5 wt % DTSACL	AP-*grafted*-5%DTSACL	166.6; 0.1794	
PBA_0.8_-*co*-PNMA_0.2_ + 10 vol % DTSACL	AP-*grafted*-10%DTSACL	333.4; 0.3586	
PBA_0.8_-*co*-PNMA_0.2_ + 0 wt % DTSACL + 1 wt % CHG	AP-*grafted*-1%CHG		100.0; 0.0358
PBA_0.8_-*co*-PNMA_0.2_ + 1 wt % DTSACL + 1 wt % CHG	(AP/1%CHG)-*grafted*-1%DTSACL	33.4; 0.0358	100.0; 0.0358
PBA_0.8_-*co*-PNMA_0.2_ + 3 wt % DTSACL + 1 wt % CHG	(AP/1%CHG)-*grafted*-3%DTSACL	100.0; 0.1076	100.0; 0.0358
PBA_0.8_-*co*-PNMA_0.2_ + 5 wt % DTSACL + 1 wt % CHG	(AP/1%CHG)-*grafted*-5%DTSACL	166.6; 0.1794	100.0; 0.0358
PBA_0.8_-*co*-PNMA_0.2_ + 10 wt % DTSACL % CHG	(AP/1%CHG)-*grafted*-10%DTSACL	333.4; 0.3586	100.0; 0.0358

### Preparation of (AP/*b*%CHG)-*grafted*-*a*%DTSACL in the Film Form

The fabrication processes of the (AP/*b*%CHG)-*grafted*-*a*%DTSACL thin film are similar to those of the bulk modified polymers above. The difference lies in the addition of an 8 times dilution process to obtain a blended compound with a solid content of about 50 mg mL^–1^, and the ready solution was spun-cast onto glass or 320 nm thickness silica wafer as the substrate with a spinning speed of 1000 rpm for 60 s. Then, we proceeded with the same heating process.

### Antimicrobial Activity of (AP/*b*%CHG)-*grafted*-*a*%DTSACL against *E. coli* and *S. aureus*

#### For Bactericidal Efficiency Measurement

The American Association of Textile Chemists and Colorist (AATCC-100) method has been adopted in this study to calculate the bactericidal efficiency of (AP/*b*%CHG)-*grafted*-*a*%DTSACL,^74,75^ by mixing such blended compounds in a bacterial suspension prepared in a neutralizing solution with two different dilution concentrations of 10^3^ for the *E. coli* (ATCC 25922) test and 10^4^ CFU mL^–1^ for *S. aureus* (ATCC 21351) at room temperature. These suspensions were left for 18 h under the incubation conditions. The bactericidal efficiency of (AP/*b*%CHG)-*grafted*-*a*%DTSACL was calculated using the following formula
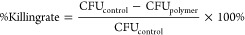
1Here, CFU_polymer_ is the number of surviving bacteria and CFU_control_ is the number of growing bacteria on the specified nutrients without the addition of AP or antimicrobial agent blends.

#### For the Inhibition Zone Test

Various concentrations of (AP/*b*%CHG)-*grafted*-*a*%DTSACL were prepared for a series of antimicrobial assays that followed the previous procedure performed by Lee et al.^76^ The spread plate technique was employed for counting viable cells in bacterial solutions. Activated Gram-negative *E. coli* and Gram-positive *S. aureus* were cultured into a Luria broth (LB) and a Tryptic soy broth (TSB) nutrient medium, respectively, under the incubation condition at 36 ± 1 °C for 16 h on a rotary shaker at 200 rpm. The bacterial suspension was collected by centrifugation, washed two times with PBS buffer (PH = 7.4), and then resuspended in PBS again to adjust the cell concentration of approximately 10^9^ colony-forming units per mL (CFU mL^–1^). 0.1 mL of active bacterial culture was spread on nutrient agar plates before the sample (AP/*b*%CHG)-*grafted*-*a*%DTSACL coated on a 1 × 1 cm^2^ square glass surface substrate was overlaid on a bacterial cell-plated agar surface. The plates were incubated again under dark conditions at 36 ± 1 °C for 3 days. The length of incubation time is based on the life span of the bacteria, which can only survive for 3 days. Finally, the distance of the clear zone around the glass substrate before/after being coated with the modified polymers in the *E. coli* and *S. aureus* plates was measured.

All of the experiments were repeated three times.

### Antifungal Assessment

A preliminary assessment of the antifungal property of antimicrobial blend compounds was carried out using representative (AP/1%CHG)-*grafted*-3%DTSACL. A 50 mg mL^–1^ portion of the modified polymer was coated on the surface of the silica wafer, embedded in the bread, and then left for 21 days. Evaluation of the durability of (AP/1%CHG)-*grafted*-3% DTSACL as an antimicrobial protective film in humid conditions was carried out by monitoring the extension of the shelf life of bread. A sponge-textured white bread was allowed to soak in 25 mL of (AP/1%CHG)-*grafted*-3% DTSACL solution (solid content of 50 mg mL^–1^) and then dried at room temperature for the formation of an antimicrobial protective layer. Another piece of white bread without any treatment was also prepared as the control sample. Each white bread was sprayed with 5 mL of DI water to provide moisture and accelerate fungal growth, transferred to a sterile plastic bag, and then stored for 14 days.

## Supporting Information Available

^1^H NMR, TGA, FTIR, DSC, XPS, and FE-SEM analyses; tear-resistance and self-recoverability measurements; and the antibacterial tests of the copolymer and blended compounds (PDF)
