# Supplementary material for: Elastic and Self-Healing Copolymer Coatings with Antimicrobial Function
Source: ACS Appl Mater Interfaces. 2024 Apr 29;16(19):25194–209. doi: 10.1021/acsami.4c00431 (PMC11103657; doi:10.1021/acsami.4c00431)
Supplement: Supplementary file 1 — am4c00431_si_001.pdf [file am4c00431_si_001.pdf]

Supporting Information

Elastic and Self-Healing Copolymer Coatings with Antimicrobial Function

Livy Laysandra[‡ a], Randy Arthur Rusli[‡ a], Yu-Wei Chen[a], Shi-Ju Chen[b], Yao-Wei Yeh[c], Tsung-Lin Tsai*[c,d], Jui-Hsiung Huang[e], Kao-Shu Chuang[e], Andreas Njotoprajitno[a], and Yu-Cheng Chiu*[a,f]

[‡]Equally contributed to the work

[a]Department of Chemical Engineering, National Taiwan University of Science and Technology, Taipei City 10607, Taiwan

[b]Taipei Municipal Zhongshan Girls High School, Taipei, 10617, Taiwan

[c]Department of Biomedical Engineering, College of Engineering, National Cheng Kung University, Tainan 704, Taiwan

[d]Department of Oncology, National Cheng Kung University Hospital, College of Medicine, National Cheng Kung University, Tainan 704, Taiwan

[e]Department of Green Material Technology, Green Technology Research Institute, CPC Corporation, Kaohsiung City 811, Taiwan

[f]Advanced Research Center for Green Materials Science and Technology, National Taiwan University, Taipei, 10617 Taiwan

*Corresponding Authors

E-mail: sloantsai@mail.ncku.edu.tw; ycchiu@mail.ntust.edu.tw

List of Contents for the Supporting Information

S1. ^{1}H NMR analyses of the modified polymer 2

S2. TGA measurements 3

S3. Cross-linking Study 4

S4. Tear resistance Study 6

S5. Investigation of possible covalent and non-covalent interactions in commercial cationic antimicrobial agent mixtures DTSACL/CHG 7

S6. Self-Recoverability Study 8

S7. DSC analysis 9

S8. XPS and EDX analyses 10

S10. Antifungal Assessment 14

S11. Water contact angle tests 15

S12. Table Comparison 16

S13. References 20

S1. ^{1}H NMR analyses of the modified polymer

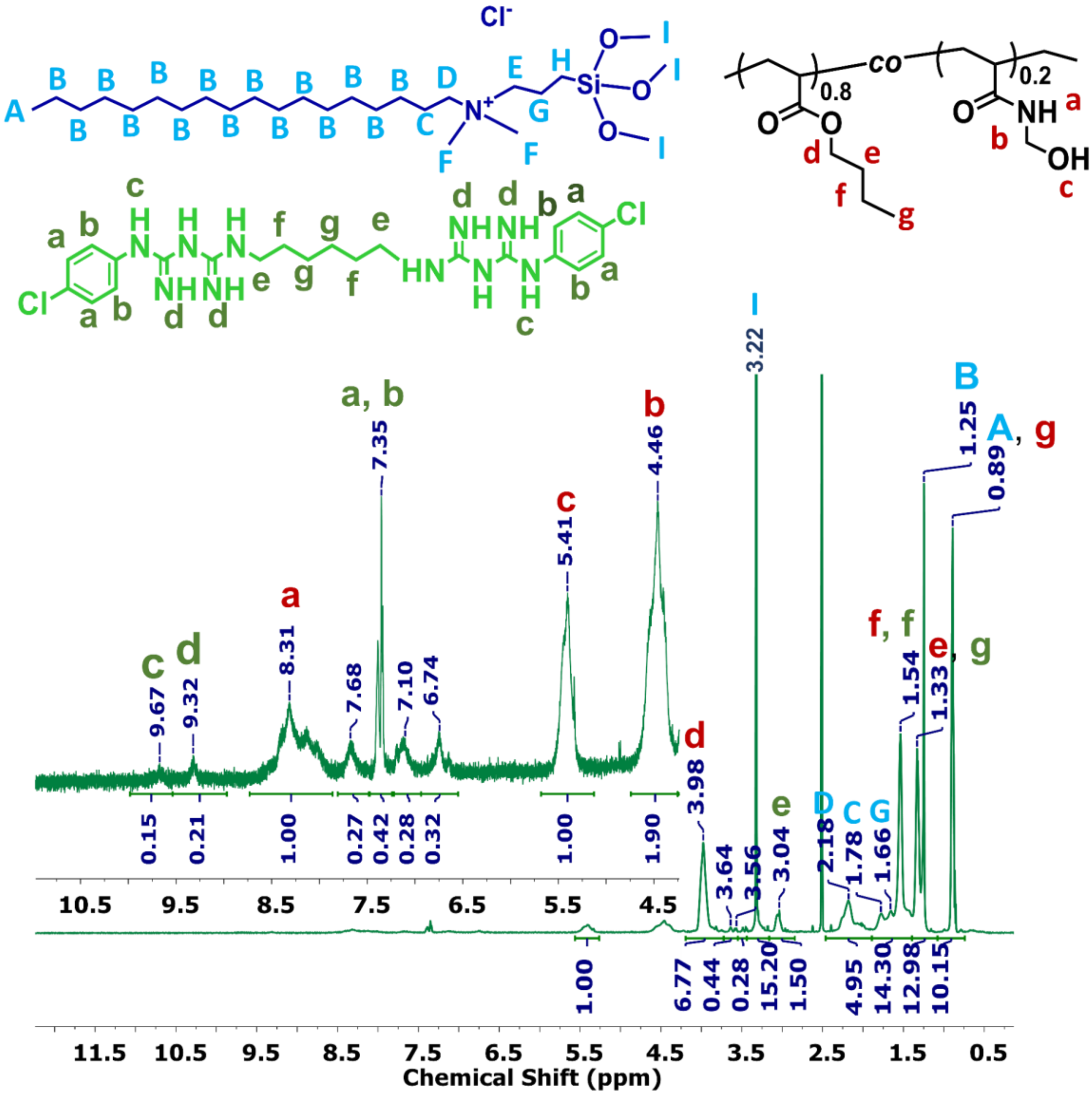

Figure S1. ^{1}H NMR spectrum of (AP/1%CHG)-*grafted*-3%DTSACL as a representative sample in DMSO_d$_6$ and their chemical structures with peaks assignments.

S2. TGA measurements

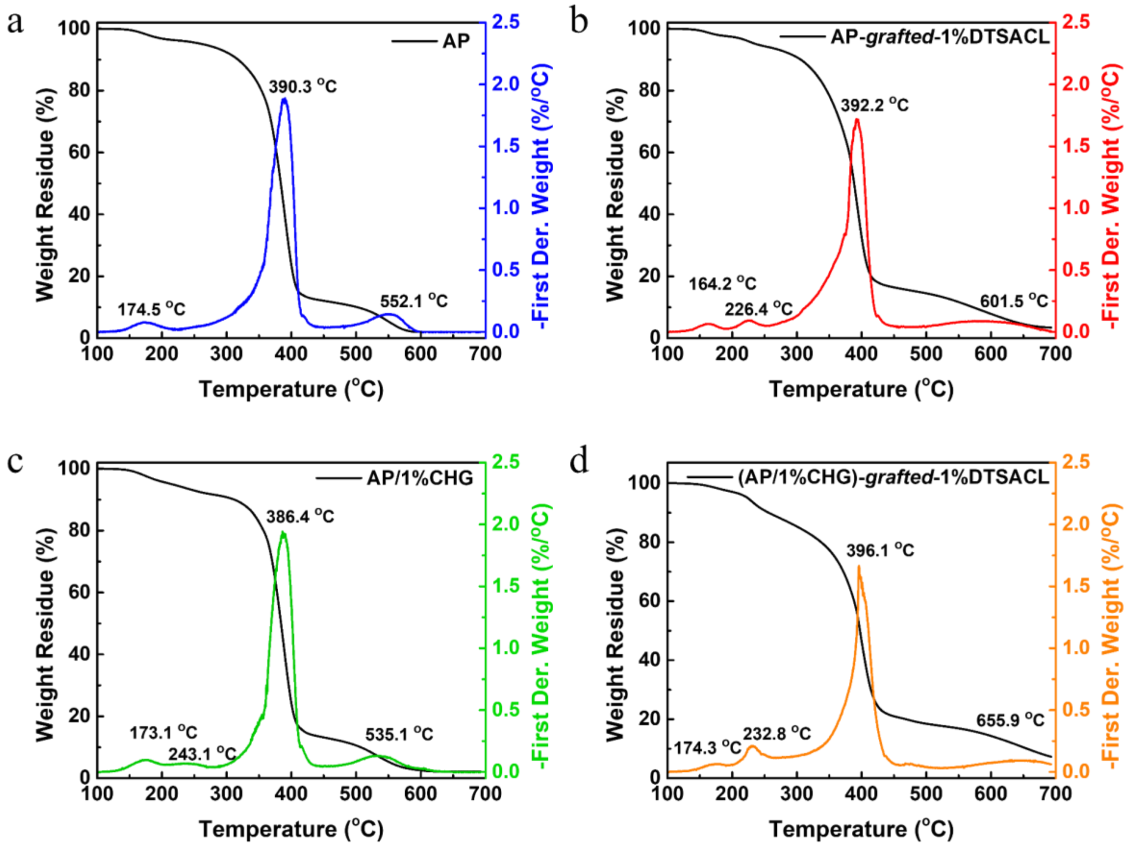

Figure S2. The influence of antibacterial agents on the thermal property of the AP system was investigated via TGA and the corresponding DTGA curves: (a) AP; (b) AP-*grafted*-1%DTSACL; (c) AP/1%CHG; and (d) (AP/1%CHG)-*grafted*-3%DTSACL films. All of the proposed samples were thermally stable in the N_2 atmosphere without noticeable weight loss until 150 °C. The decomposition temperature of AP; AP-*grafted*-1%DTSACL; AP/1%CHG; and (AP/1%CHG)-*grafted*-3%DTSACL at 5% weight loss in TGA were 263.3, 237.7, 215.6, and 223.9 °C, respectively.

S3. Cross-linking Study

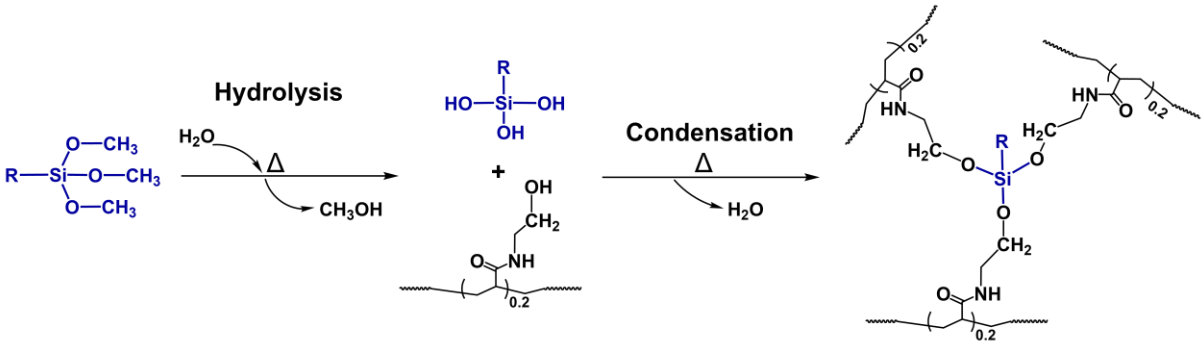

Figure S3. Hydrolysis and condensation reactions associated with the trimethoxy silane group in DTSACL covalently bonds with the reactive hydroxyl group in the NMA side chain, which leads to the formation of AP-*grafted-a*%DTSACL thin film. In this case, a trace amount of water comes from the moisture adsorbed during the solvent elimination process at room temperature or comes from the silane solution mixed into the polymer. The presence of this water triggers the hydrolysis process for the trimethoxy silane group in DTSACL.

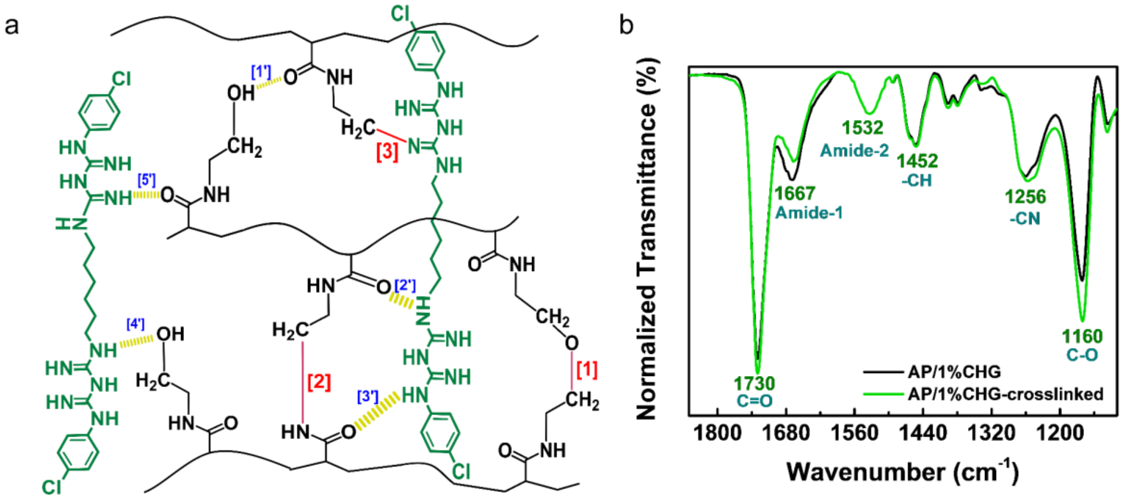

Figure S4. (a) Schematic illustration of the possible synergistic effect in the cross-linking reaction on the blended 1% CHG with AP. Three types of covalent bonding formations were proposed: [1] represents the dehydration of the hydroxyl group on the NMA segment formed bis(methylene ether) by the loss of OH^- and H^+; [2] represents the formation of a methylene bridge by the loss of formaldehyde between NMA side chains; and [3] represents the covalent linking of C–N= groups from the CHG structure with the NMA side chains. Furthermore, various types of non-covalent hydrogen interaction [1'] between NMA side chains and [2'–5'] between the NMA side chain and the two biguanide groups of CHG were also presented. (b) Enlargement from **Figure 1d** of normalized ATR-FTIR spectrum focusing on the range wavenumber of 1100 – 1850 cm^{-1}.

Supporting Discussion:

Figure S4a shows the potential for the formation of a large number of covalent and non-covalent hydrogen bond networks capable of developing complex structures in the crosslinked modified polymer. FTIR spectroscopy was performed to demonstrate that the resultant thermally induced crosslinking reactions formed covalent and non-covalent interactions between the NMA side chains and between the NMA side chains and the CHG compound. As depicted in **Figure S4b**, the strong peaks at 1667 cm^{-1} and 1532 cm^{-1} are assigned to the amide I and amide II bands, respectively, which correspond to the amide groups of both the NMA side chain and the biguanide groups of CHG. Integral area reduction on Amide-I implies the =NH breakage of the biguanide group and the enhancement integral area at peak 1256 cm^{-1} confirming the covalent bond formation of the C–N– group.[1] Meanwhile, the expansion of the integral area belonging to the C–O–C peak is well defined as the successful covalent bonding formation focusing on the AP system.[2] It was associated with the damage of hydroxy groups with the loss of OH^- and H^+ accompanied by the formation of bis(methylene ether) between the NMA segments.[3]

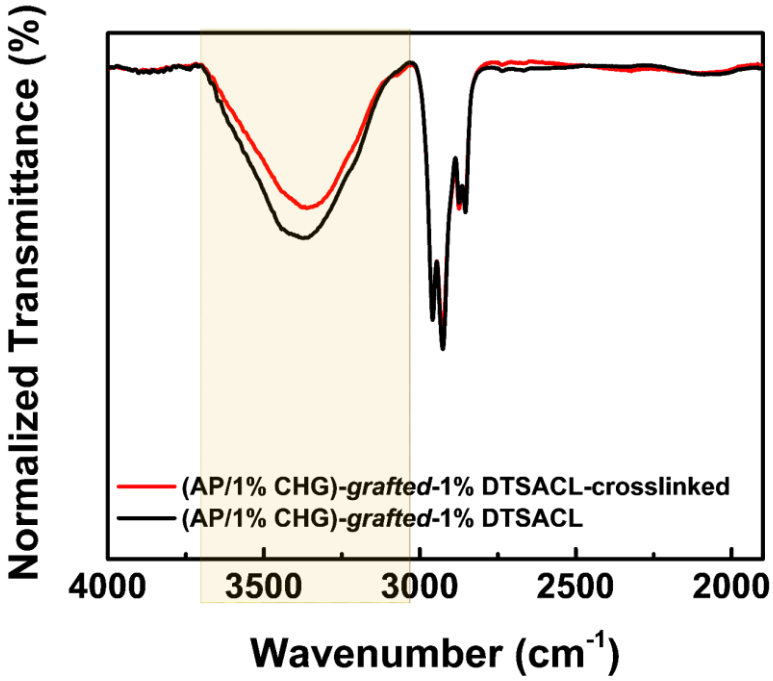

Figure S5. Normalized ATR-FTIR spectrum of (AP/1%CHG)-*grafted*-1%DTSACL before and after thermal-triggered cross-linking reaction that focusing on the range wavenumber of 1900 – 4000 cm^{-1}.

S4. Tear resistance Study

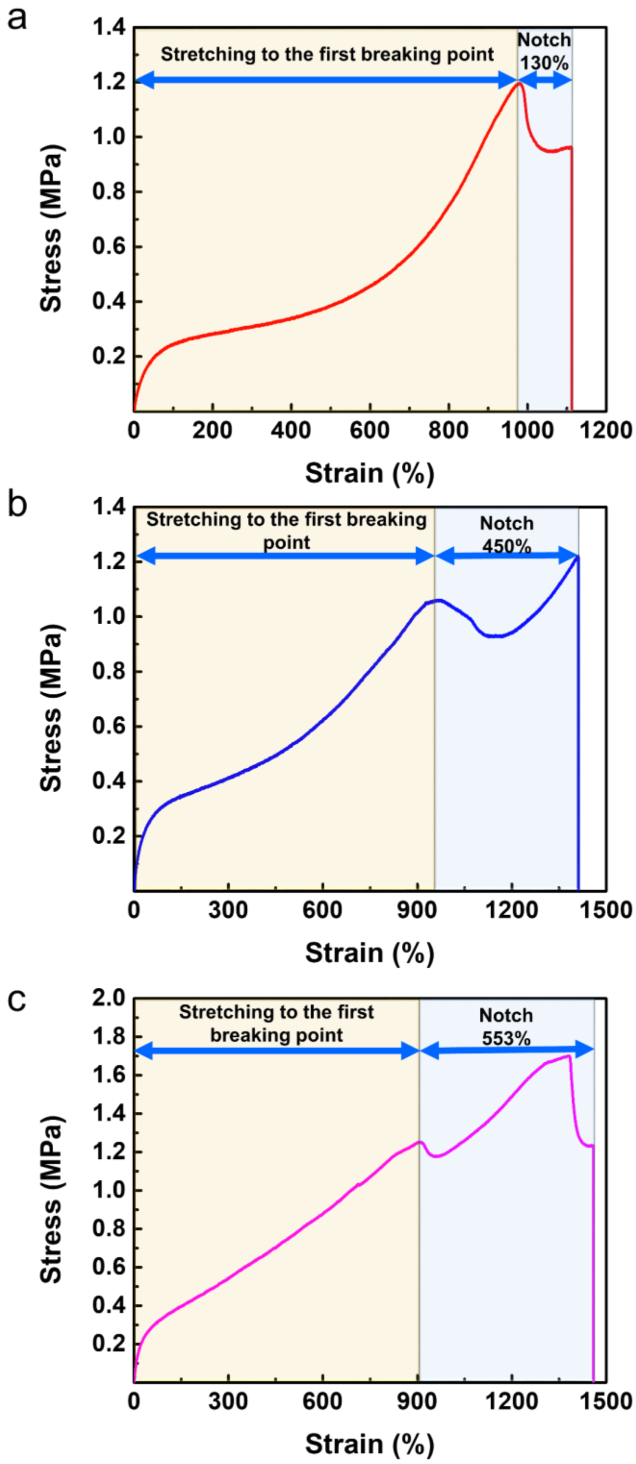

Figure S6. Close examination of tear-resistance begins when a slight fracture is detected during the tensile test for the modified polymers of (a) (AP/1%CHG)-*grafted*-3%DTSACL, (b) (AP/1%CHG)-*grafted*-5%DTSACL, and (c) (AP/1%CHG)-*grafted*-10%DTSACL.

S5. Investigation of possible covalent and non-covalent interactions in commercial cationic antimicrobial agent mixtures DTSACL/CHG

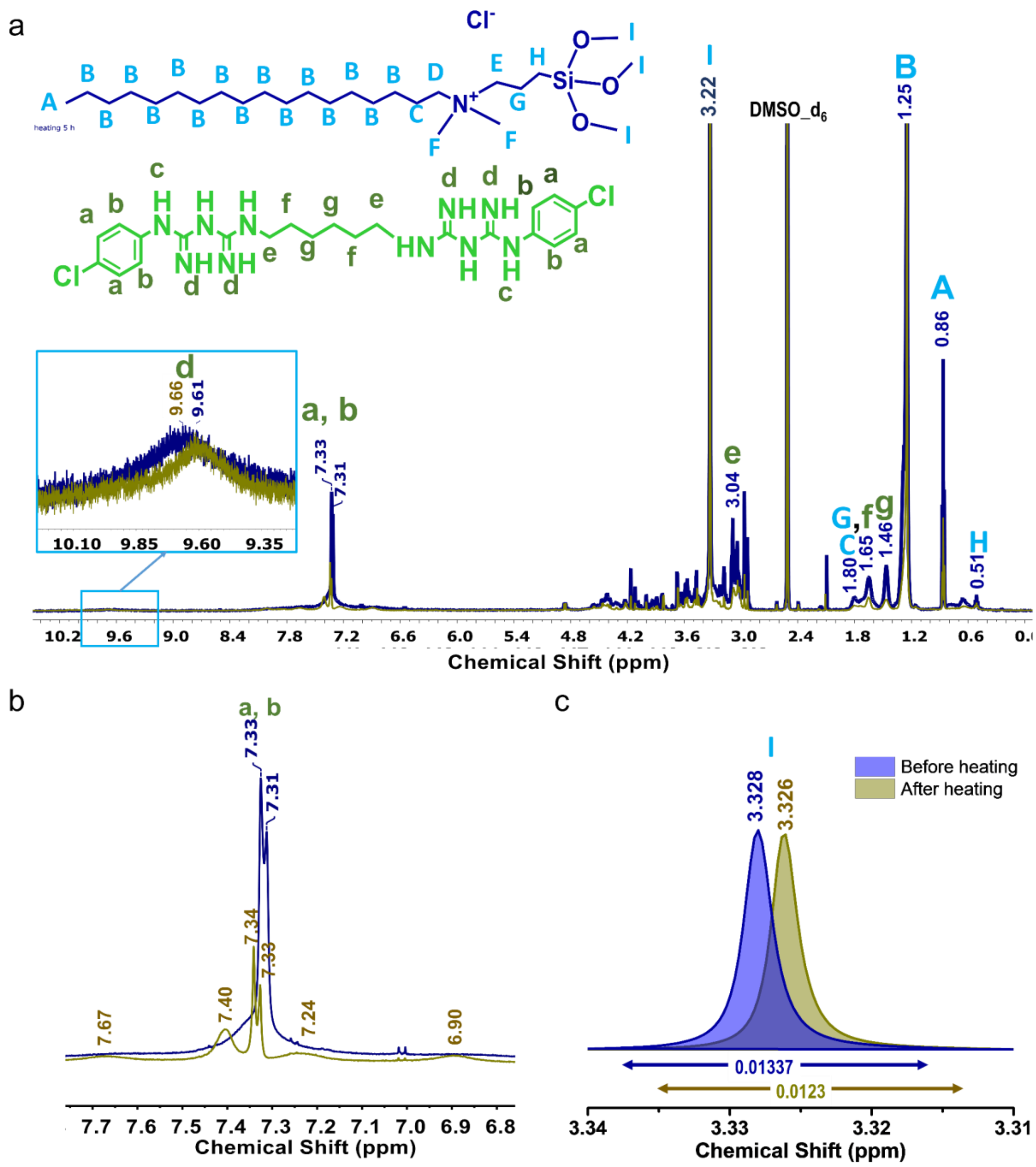

Figure S7. ^{1}H NMR spectra in DMSO_d$_6$ of the DTSACL/CHG (1:1 *v/v*) mixture before (navy blue line) and after (grayish-green line) heating at 100 °C for 3 h. (a) Display of overall ^{1}H NMR analysis results and magnification in the range of 9.20 - 10.20 ppm. (b) Focus on the range of 6.75 - 7.72 ppm. (c) Focus on the range of 3.31 - 3.34 ppm with peak intensity values estimated based on integral spectrum area which was first normalized by the DMSO_d$_6$ solvent intensity of the specimen in question, followed by integrating the area of the region of interest and the results were compared.

S6. Self-Recoverability Study

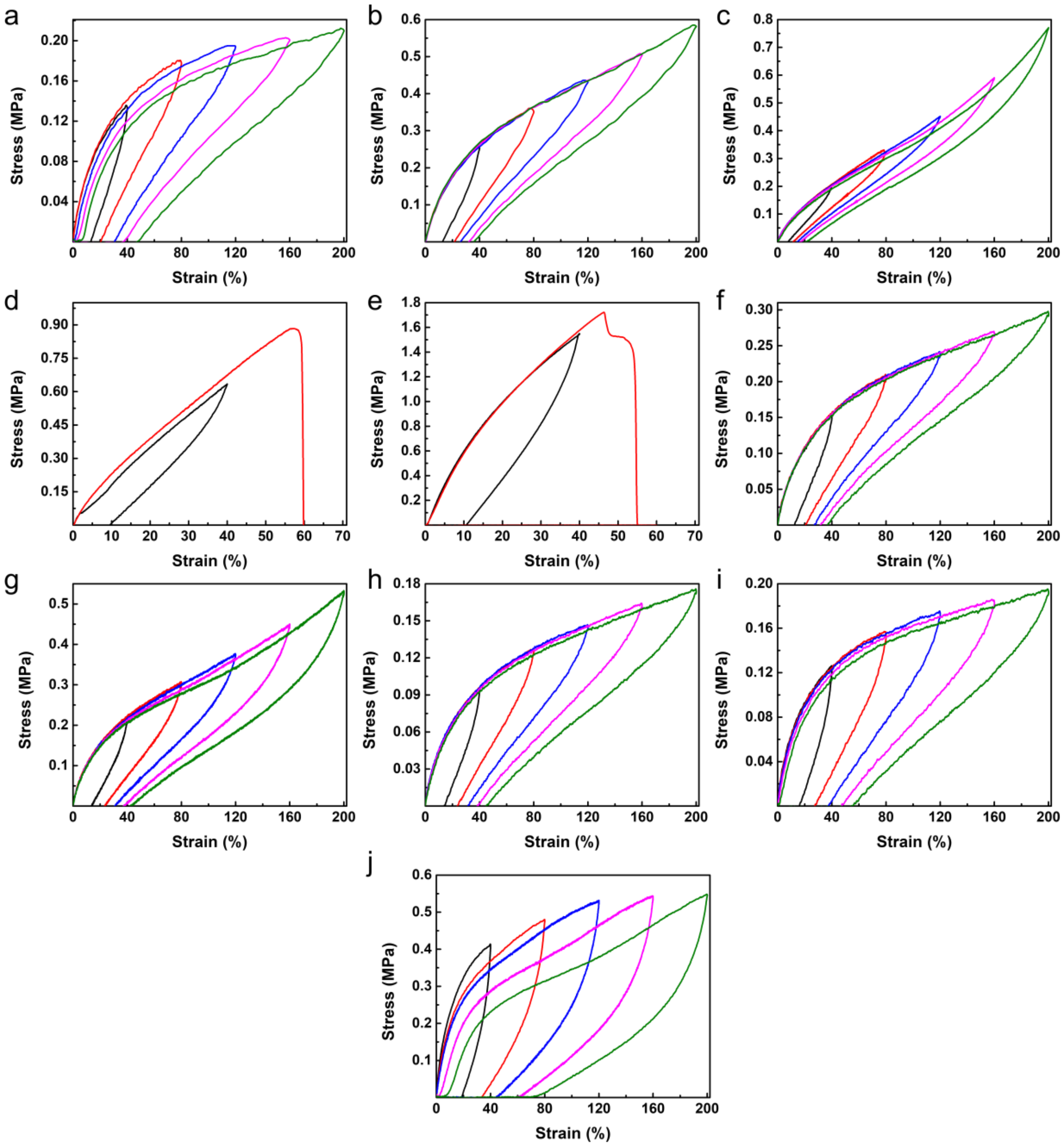

Figure S8. Representative cyclic tensile loading-unloading curves with gradual strain increase from 40 to 200% strain for the: (a) AP; (b) AP-*grafted*-1%DTSACL; (c) AP-*grafted*-3%DTSACL; (d) AP-*grafted*-5%DTSACL; (e) AP-*grafted*-10%DTSACL; (f) AP/1%CHG; (g) AP/1%CHG-*grafted*-1%DTSACL; (h) (AP/1%CHG)-*grafted*-3%DTSACL; (i) (AP/1%CHG)-*grafted*-5%DTSACL; and (j) (AP/1%CHG)-*grafted*-10%DTSACL. The pause time at the change of maximum strain loading is 5 min.

S7. DSC analysis

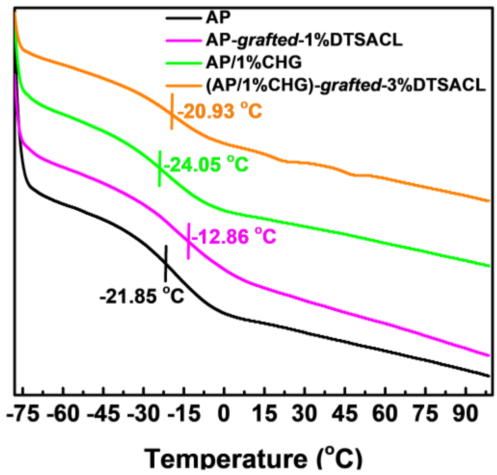

Figure S9. The DSC measurement of AP and modified polymers after subjected to the heating process.

S8. XPS and EDX analyses

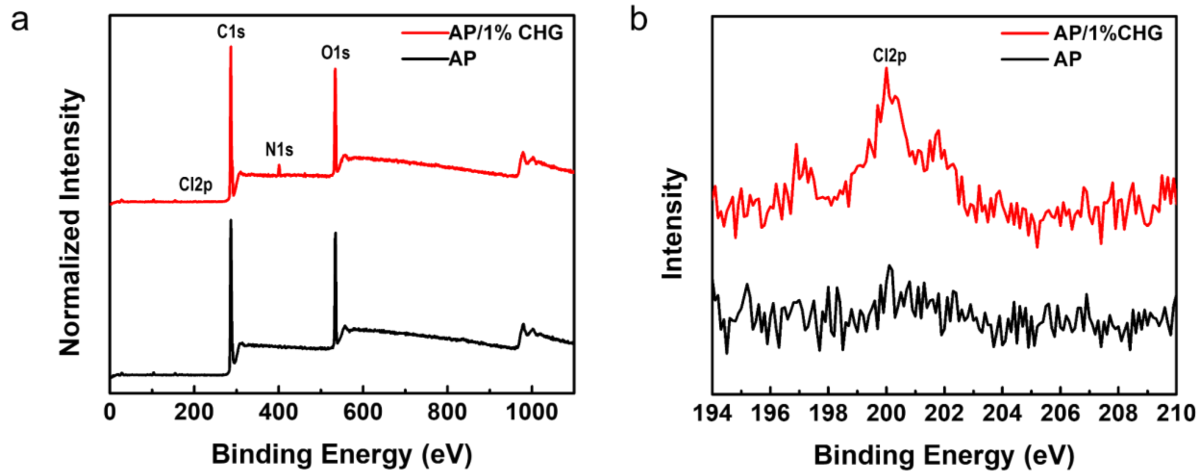

Figure S10. XPS survey scan spectra of AP and AP/1%CHG where: (a) full-scan and (b) focus on Cl2p element.

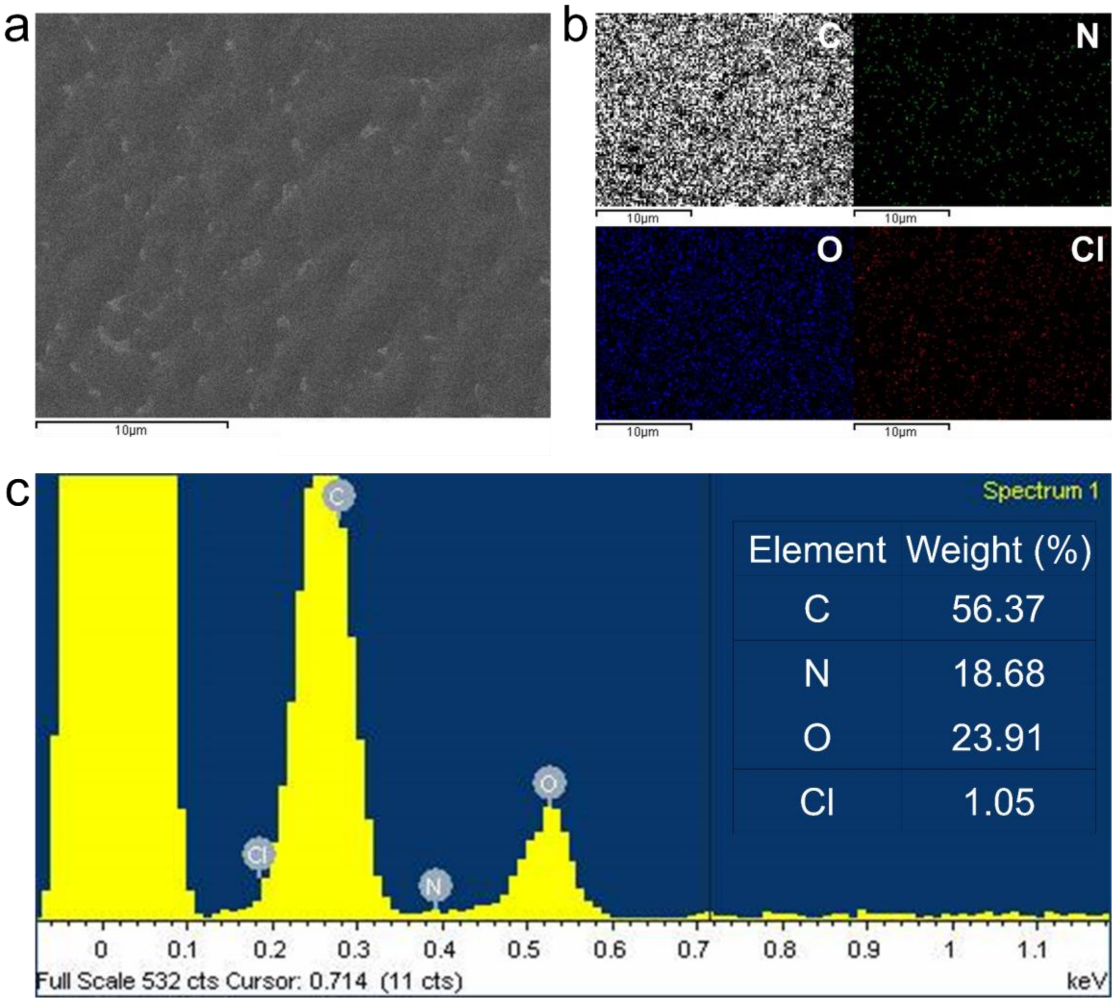

Figure S11. (a) FE-SEM image of AP/1%CHG thin film, (b) the elemental mapping image of carbon (C), nitrogen (N), oxygen (O), and chlorine (Cl) confirming the proper distribution of CHG embedded into the AP and (c) corresponding EDX spectra of AP/1%CHG.

S9. Antibacterial Properties

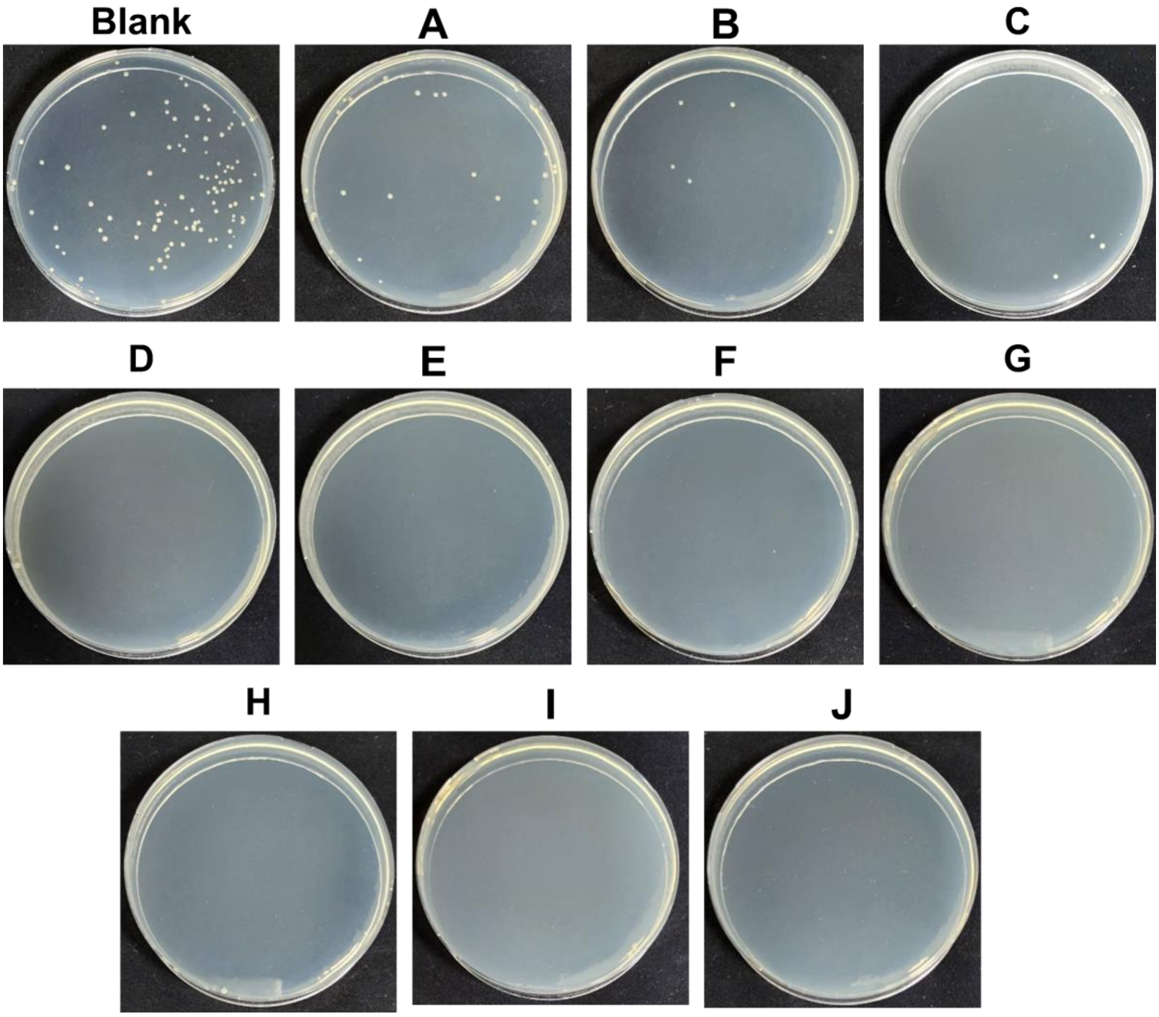

Figure S12. Digital images of surviving bacteria on AP and modified polymers after being co-cultured with *E. coli*. The bacterial concentration is 10^3 CFU mL^{-1}. The description of each photograph is as follows: A = Blank without any additional sample; B = $PBA_{0.8}$-*co*-$PNMA_{0.2}$ or AP; C = AP-*grafted*-1%DTSACL; C = AP-*grafted*-3%DTSACL; D = AP-*grafted*-5%DTSACL; E = AP-*grafted*-10%DTSACL; F = AP/1%CHG; G = (AP/1%CHG)-*grafted*-1%DTSACL; H = (AP/1%CHG)-*grafted*-3%DTSACL; I = (AP/1%CHG)-*grafted*-5%DTSACL; and J = (AP/1%CHG)-*grafted*-10%DTSACL.

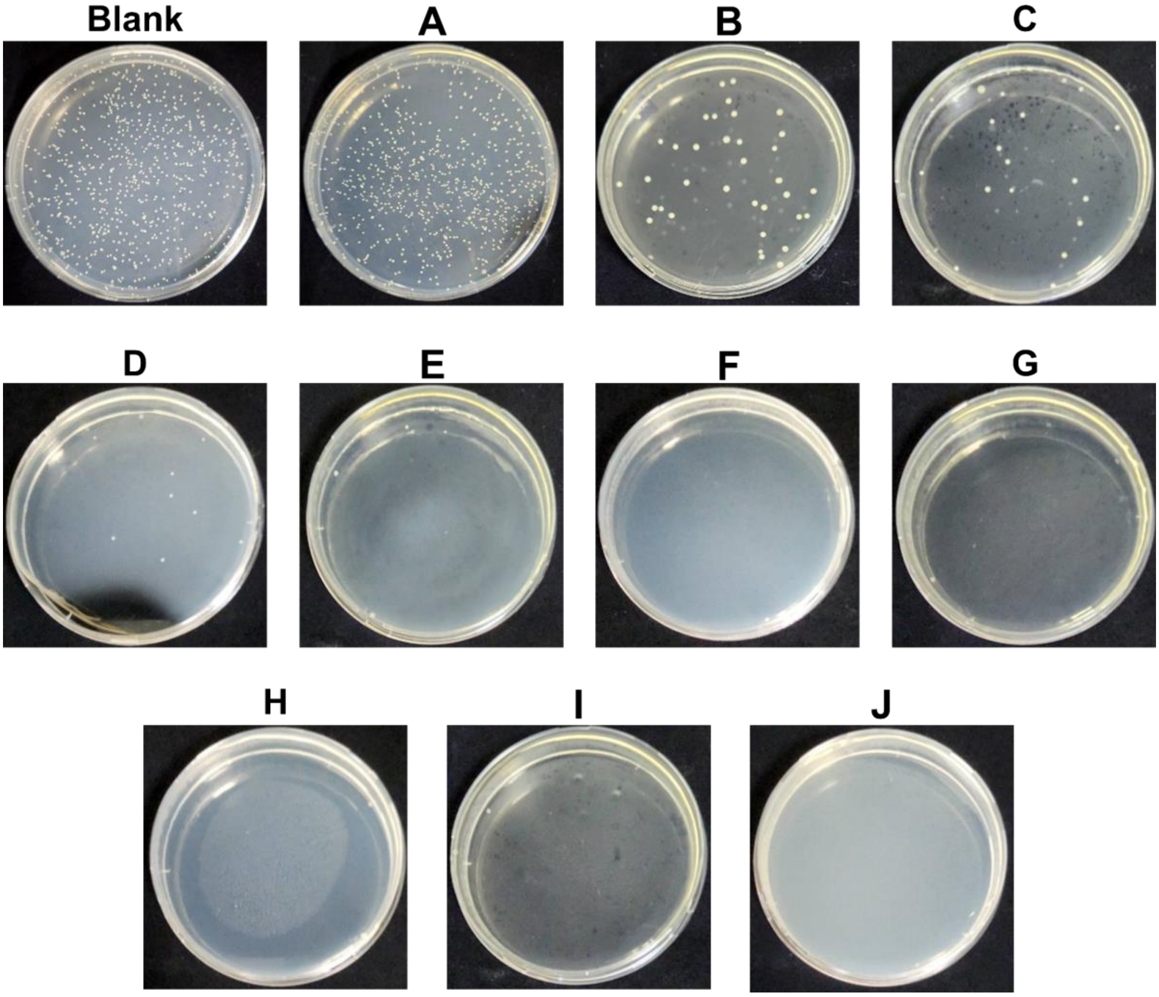

Figure S13. Digital images of surviving bacteria on AP and modified polymers after being co-cultured with *S. aureus*. The bacterial concentration is 10^4 CFU mL^{-1}. The description of each photograph is as follows: A = Blank without any additional sample; B = $PBA_{0.8}$-*co*-$PNMA_{0.2}$ or AP; C = AP-*grafted*-1%DTSACL; C = AP-*grafted*-3%DTSACL; D = AP-*grafted*-5%DTSACL; E = AP-*grafted*-10%DTSACL; F = AP/1%CHG; G = (AP/1%CHG)-*grafted*-1%DTSACL; H = (AP/1%CHG)-*grafted*-3%DTSACL; I = (AP/1%CHG)-*grafted*-5%DTSACL; and J = (AP/1%CHG)-*grafted*-10%DTSACL.

S10. Antifungal Assessment

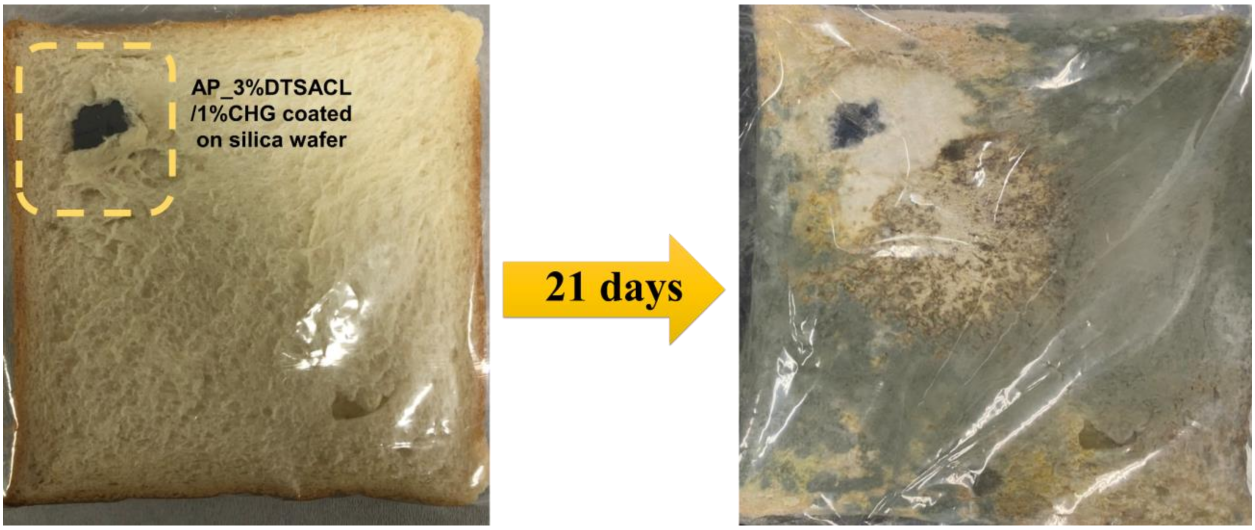

Figure S14. Appearance changes from the first to the 21 days of bread stored under cling wrap conditions at room temperature. There is no special treatment of bread before use. (AP/1%CHG)-*grafted*-3%DTSACL-coated silica wafer was embedded in the upper left corner of the bread.

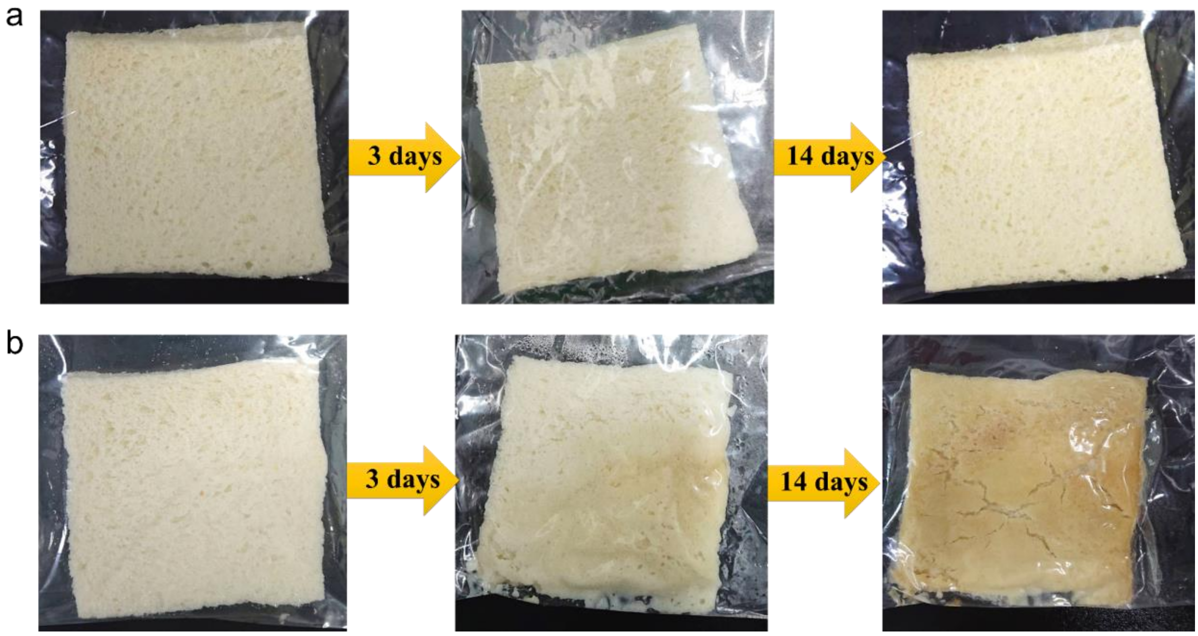

Figure S15. Visual appearance of the white bread stored (a) with and (b) without antimicrobial treatment from day 1 to day 14.

S11. Water contact angle tests

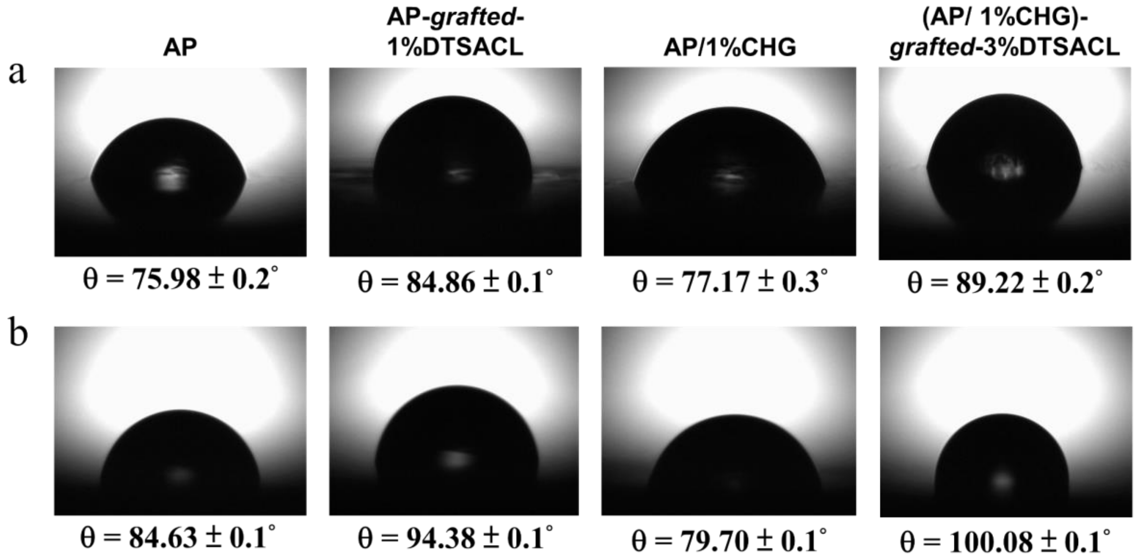

Figure S16. Images of the water contact angles (WCA) test results of AP, AP-*grafted*-1%DTSACL, AP/1%CHG, and (AP/1%CHG)-*grafted*-3%DTSACL (a) before and (b) after cross-linking at 100 °C for 3 h.

S12. Table Comparison

Table S1. Summary of practical applications antimicrobial substances as coatings.

Resource	Material	Application	Ref
US6866936B2	Phosphorylcholine	Medical devices, implants, drug delivery systems	[4]
US8728508B2	Polyurethanes, silicone elastomers, and hydrogels.	Implantable medical devices	[5]
US8871869B2	Silicone-based polymers, fluorinated polymers, polyurethanes.	Marine coatings	[6]
WO1998058990A1	Hydrophobins	Medical devices, food processing equipment, water treatment systems, and marine coatings	[7]
US20050266038A1	Heparin	Implantable medical devices	[8]

Table S2. Evaluation of the biocompatibility of each material used in this study based on other reported studies.

Polymer	Cytotoxicity test		Ref.
	Method	Results	
PBA	Cytotoxicity assay according to slight modification of ISO-10993-5 and USP24 on L929 cells (2.2×10^4 cells/dish) for 72 h of incubation.	No detectable toxic zone around or under specimen.	9
P(HEMA-*co*-BA) with BA contents of 10 – 25 %		No detectable toxic zone around or under specimen.	
cPnBA0100	EN ISO 10993-5 on L929 cells (60×10^3 cells cm^{-2}) for 48 h of incubation.	The cell viability, the cellular lactate dehydrogenase release, and the activity of the mitochondrial dehydrogenases were not different from the results on reference polystyrene as the control sample, assessing the sample as non-cytotoxic.	10
P(BA-*co*-MMA)	Cell viability measured by MTT assay on human keratinocytes cells (HaCaT) for 24 and 96 h of incubation.	The copolymer film was not cytotoxic, owing to the cell viability after 24 h was 94% and even increased to 138% after 96 h.	11
P(NMA-*co*-DAA) hydrogel	Cell counting kit-8 (CCK-8) assay on CT-26 cells for 24 and 48 h of incubation.	The cell viability was higher than 90% after incubation with a sterile hydrogel solution up to 0.5% for 24 and 48 h.	12
MPNN hydrogel	CCK-8 assay on bone-derived mesenchymal stem cells (BMSCs) for 24 and 72 h of incubation.	maintains high viability of BMSC cells to reach or exceed 100% after 24 – 72 h of incubation.	13

PBA : Homopolymer n-butyl acrylate
P(HEMA-*co*-BA) : Copolymer of 2-hydroxymethyl methacrylate with n-butyl acrylate
cPnBA0100 : Poly(butyl acrylate) crosslinked with 0.4 wt% poly(propylene glycol) dimethacrylate (PPGDMA).
P(BA-*co*-MMA) : Copolymer of butyl acrylate with methyl methacrylate.
P(NMA-*co*-DAA) : Copolymer of N-hydroxymethyl acrylamide with diacetone acrylamide.
MPNN hydrogel : Dexamethasone (Dex)-loaded MXene-poly(N-isopropylacrylamide)-*co*-N-(Hydroxymethyl) acrylamide hydrogel.
MTT assay : 3-(4,5-dimethylthiazol-2-yl)-2,5-diphenyl-2H-tetrazolium bromide, a method for Error Minimization and Interpretation in Measuring Cytotoxicity and Estimating Cell Viability.

Table S3. Surface elemental areas, compositions, and functionality ratios of AP and AP/1%CHG obtained from XPS analysis.

Specimen	Area				Element (%)				Functionality ratio			
	C	N	O	Cl	C	N	O	Cl	C/C	N/C	O/C	Cl/C
AP	25,178.63	238.09	17,371.81	-	76.83	0.46	22.71	-	1	0.01	0.30	-
AP/1%CHG	25,082.55	1,444.94	17,185.74	139.61	75.10	2.72	22.04	0.14	1	0.04	0.29	0.0018

The sensitivity factor for C, N, O, and Cl elements were 0.31, 0.50, 0.73, and 0.95, respectively.

Table S4. List of prices, brands and quantities of chemicals required to synthesis $PBA_{0.8}$-*co*-$PNMA_{0.2}$ embedded with CHG and DTSACL antimicrobial agents.

Chemicals	Brand	Quantity	CAS	Price (USD)	Quantity on a laboratory scale/batch
BA	Sigma-Aldrich	1L	141-32-2	78	8 mL
AIBN	Tokyo Chemical Industry Co, Ltd	25 g	78-67-1	75	46 mg
NMA	Tokyo Chemical Industry Co	25 g	924-42-5	28	1.42 g
Methanol anhydrous	Sigma-Aldrich	1L	67-56-1	73	32 mL
DTSACL	Acros Organics	100 mL	27668-52-6	91	Depends on the target *wt*%
CHG	Chengyi Chemical Raw Materials Co., Ltd	25 kg	18472-51-0	50	Depends on the target *wt*%

S13. References

1. Thajai, N.; Jantanasakulwong, K.; Rachtanapun, P.; Jantrawut, P.; Kiattipornpithak, K.; Kanthiya, T.; Punyodom, W., Effect of chlorhexidine gluconate on mechanical and anti-microbial properties of thermoplastic cassava starch. *Carbohydr Polym* **2022,** *275*, 118690.
2. Laysandra, L.; Chuang, C.-H.; Kobayashi, S.; Au-Duong, A.-N.; Cheng, Y.-H.; Li, Y.-T.; Mburu, M. M.; Isono, T.; Satoh, T.; Chiu, Y.-C., Design of Self-Cross-Linkable Poly(n-butyl acrylate)-co-poly[N-(hydroxymethyl)acrylamide] Amphiphilic Copolymers toward Elastic and Self-Healing Properties. *ACS Appl. Polym. Mater.* **2020,** *2* (12), 5432-5443.
3. Krishnan, S.; Klein, A.; El-Aasser, M. S.; Sudol, E. D., Influence of Chain Transfer Agent on the Cross-Linking of Poly(n-butyl methacrylate-co-N-methylol acrylamide) Latex Particles and Films. *Macromolecules* **2003,** *36*, 3511-3518.
4. Opolski, M. P. Articles with Hydrophilic Coating. 2005.
5. Nielsen, B. R.; Madsen, N. J. Hydrophilic Coating and A Method for The Preparation Thereof. . 2014.
6. Dias, A. J. A. A.; Hensen, G. J. E.; Belt, J. W.; Rooijmans, M.; Bont, N. H. M. D.; Currie, E. P. K. Hydrophilic coating. 2014.
7. Madsen, N. J. Method of Preventing Adhesion of Microorganisms to Solid Surfaces Using Hydrophobin. 1998.
8. Glauser, T.; Michal, E.; Claude, C.; Pacetti, S. Antifouling Heparin Coatings. 2005.
9. Prasitsilp, M.; Siriwittayakorn, T.; Molloy, R.; Suebsanit, N.; Siriwittayakorn, P.; Veeranondha, S., Cytotoxicity Study of Homopolymers and Copolymers of 2-Hydroxyethyl Methacrylate and Some Alkyl Acrylates for Potential use as Temporary Skin Substitutes. *J. Mater. Sci.: Mater. Med.* **2003,** *14*, 595-600.
10. Cui, J.; Kratz, K.; Hiebl, B.; Jung, F.; Lendlein, A., Soft Poly(n-Butyl Acrylate) Networks with Tailored Mechanical Properties Designed as Substrates for in Vitro Models. *Polym. Adv. Technol.* **2010,** *22* (1), 126-132.
11. Rodrigues, L. D. A.; Hurtado, C. R.; Macedo, E. F.; Tada, D. B.; Guerrini, L. M.;Oliveira, M. P., Colloidal Properties and Cytotoxicity of Enzymatically Hydrolyzed Cationic Starch-graft-Poly(Butyl Acrylate-co-Methyl Methacrylate) Latex by Surfactant-Free Emulsion Polymerization for Paper Coating Application. *Prog. Org. Coat.* **2020,** *145*.
12. Shen, J.; Chang, L.; Chen, D.; Wang, Y.; Li, W.; He, Y.; Qin, J., Cross-linking Induced Thermo-Responsive Self-Healing Hydrogel with Gel-Sol–Gel Transition Constructed on Dynamic Covalent Bond. *J. Polym. Res.* **2021,** *28* (4), 132.
13. Chen, Y.; Liu, W.; Wan, S.; Wang, H.; Chen, Y.; Zhao, H.; Zhang, C.; Liu, K.; Zhou, T.; Jiang, L.; Cheng, Q.; Deng, X., Superior Synergistic Osteogenesis of MXene-Based Hydrogel through Supersensitive Drug Release at Mild Heat. *Adv. Funct. Mater.* **2023,** *34* (2), 230919.
